# Lessons from the modular organization of the transcriptional regulatory network of Bacillus subtilis

**DOI:** 10.1186/1752-0509-7-127

**Published:** 2013-11-16

**Authors:** Julio A Freyre-González, Alejandra M Manjarrez-Casas, Enrique Merino, Mario Martinez-Nuñez, Ernesto Perez-Rueda, Rosa-María Gutiérrez-Ríos

**Affiliations:** 1 Programa de Genómica Evolutiva, Centro de Ciencias Genómicas, Universidad Nacional Autónoma de México, Av. Universidad s/n,Col. Chamilpa, Cuernavaca, Morelos 62210, México; 2 Departamentos de Microbiología Molecular, Instituto de Biotecnología, Universidad Nacional Autónoma de México, Apdo. Postal 510-3, Cuernavaca, Morelos 62250, México; 3 Ingeniería Celular y Biocatálisis, Instituto de Biotecnología, Universidad Nacional Autónoma de México, Apdo. Postal 510-3, Cuernavaca, Morelos 62250, México

**Keywords:** Master regulators, σ factors, Modularity, Hierarchy, Regulatory network, Model organisms, Paralogous proteins

## Abstract

**Background:**

The regulation of gene expression at the transcriptional level is a fundamental process in prokaryotes. Among the different kind of mechanisms modulating gene transcription, the one based on DNA binding transcription factors, is the most extensively studied and the results, for a great number of model organisms, have been compiled making it possible the *in silico* construction of their corresponding transcriptional regulatory networks and the analysis of the biological relationships of the components of these intricate networks, that allows to elucidate the significant aspects of their organization and evolution.

**Results:**

We present a thorough review of each regulatory element that constitutes the transcriptional regulatory network of *Bacillus subtilis.* For facilitating the discussion, we organized the network in topological modules. Our study highlight the importance of σ factors, some of them acting as master regulators which characterize modules by inter- or intra-connecting them and play a key role in the cascades that define relevant cellular processes in this organism. We discussed that some particular functions were distributed in more than one module and that some modules contained more than one related function. We confirm that the presence of paralogous proteins confers advantages to *B. subtilis* to adapt and select strategies to successfully face the extreme and changing environmental conditions in which it lives.

**Conclusions:**

The intricate organization is the product of a non-random network evolution that primarily follows a hierarchical organization based on the presence of transcription and σ factor, which is reflected in the connections that exist within and between modules.

## Background

*Bacillus subtilis* is the best-characterized member of the Gram-positive bacteria and represents an excellent model for the study of gene regulation and metabolism in the Firmicute phylum. This Gram-positive bacterium is a facultative aerobe that was initially classified as a soil bacterium, but its ability to grow in many diverse terrestrial and aquatic environments, from the root surface of some plants to the gastrointestinal tract of some animals, is now well known. This ability to adapt to different environments has been mainly attributed to the formation of spores, which occurs under certain conditions of stress and nutrient scarcity.

Many characteristics of this bacterium have been elucidated through the study of its complete genome. A sequence analysis of its genome revealed the presence of more than 120 transcription regulatory proteins (including 14 σ factors) that regulate the expression of 1,475 promoters [1]. This knowledge provides useful information to construct the *Bacillus subtilis* Transcriptional Regulatory Network (TRN) and make a deep and careful discussion of the intricate and elegant design that *B. subtilis* displays in the microbial world. In addition, in this bacterium, global and local regulators are integrated, which allows the TRN to be rewired in response to metabolic signals, spore formation and germination processes. This rewiring is mediated by the interplay of transcription factors (TFs) and σ factors that have fundamental roles in regulating important and well-defined metabolic and development processes, such as sporulation and germination.

An essential aspect of this work is the manual curation of the information and a thorough review of each regulatory element that constitutes the transcriptional regulatory network of *Bacillus subtilis.* As a way of presenting the vast amount of information published on *B. subtilis,* we organized this data onto network topological modules, in such a way that the relationship of different regulatory elements and the role that they play in the *B. subtilis* metabolism could be analyze from a more integrated point of view as done for other networks [2,3]. For this purpose, and based on the experimentally defined regulatory interactions of TFs and σ factors with their corresponding target gene [1], we evaluated the statistical properties of the *B. subtilis* TRN. As it has been reported for other model organisms [4-7], our results indicate that TRN of *B. subtilis* is a scale-free network with hierarchical properties consisting of nine regulatory modules that could be associated with well-defined biological processes. In addition, we discuss the evolutionary and functional implications of the topology of the TRN in this bacterial model.

## Results and discussion

### Topological organization of the *B. subtilis* regulatory network and its comparison with other TRNs

**Figure 1:**
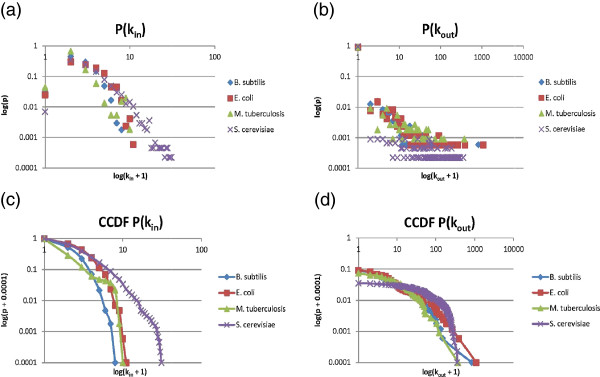
**The in and out connectivity of the *B. subtilis *TRN and other model organisms.** The graphs in **a** and **b** show the incoming (*Pin*) and outcoming (*Pout*) degree of *B. subtili*, *E. coli*, *M. tuberculosis* and *S. cerevisiae*, respectively. **c-d** shows the log-log cumulative complementary distribution (CCDF). The figures also show a comparisons of the coefficient of determination (R^2) for each regression as an indicator of the goodness of fit of the power-law model and the comparison of each of them against the R^2 for a corresponding exponential fit.

To evaluate the statistical properties and modular organization of the *B. subtilis* TRN, we obtained all of the regulatory interactions reported in DBTBS, a database of transcriptional regulation in *B. subtilis*[1]. Based on this information, we constructed a regulatory network composed of 1,626 nodes and 3,096 edges, with an average clustering coefficient of 0.538. The *P(k)* follows a power law distribution with a power-law exponent of approximately -2.11. These results are characteristic of a large, scale-free network with a modular hierarchic organization. These properties are common in other previously described regulatory networks, such as the TRN of *Escherichia coli* K12 and *Sacharomyces cerevisiae*[6,8-10]. As a point of comparison with other reported TRNs we calculated the incoming (*Pin*) and outcoming (*Pout*) degree distributions of three well-annotated prokaryotes (*B. subtilis*, E*. coli* and *M. tuberculosis*) and an eukaryote (*S. cerevisiae*), (Figures 1 a-b respectively). We observed that the *Pin* distributions are characteristic of TRN, which do not show a long tail for any organism (Figure 1a). In order to estimate the exponent for each power-law we computed the log-log cumulative complementary distribution (CCDF) and then fitted a straight-line to it using least squares (Figures 1 c-d). Additionally, we computed the coefficient of determination (R^2) for each regression as an indicator of the goodness of fit of the power-law model, and then compared each of them against the R^2 for a corresponding exponential fit. We found that the *Pout* distributions for the three bacteria are better explained by a power-law than by an exponential fit. Conversely, *S. cerevisiae Pout* distribution is better explained by an exponential distribution, so we do not computed the power-law exponent.

**Figure 2:**
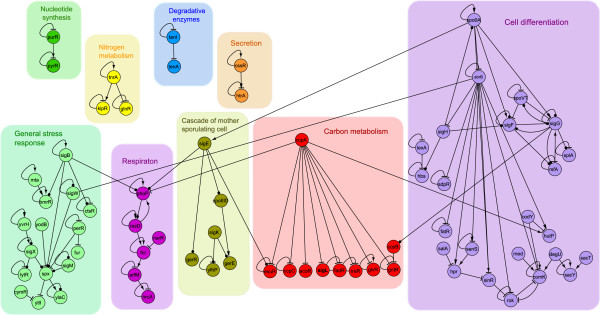
**Cross-talk between modules.** Master regulators (hubs) interconnect functional modules. At the higher levels, each master regulator is indicated. The color of each TF relates it to the module to which it belongs. We also show (top left) four disconnected groups that represent modules that are not interconnected by master regulators.

In a posterior step, we extracted a sub-network consisting of only the regulatory interactions of all known *B. subtilis* TFs and σ factors (54 and 16, respectively). We excluded σA interactions from this sub-network because, as a housekeeping factor, σA is tightly connected to almost every node of the network (with an outcoming connectivity of 782, connecting 46.5% of the genes in the network), generating a mega-module that encompasses all the basic physiological functions described in *B. subtilis*. Our resulting TRN was composed of 71 nodes and 81 edges and is supported by strong experimental evidence. The data were used as input to perform a hierarchical agglomerative average linkage clustering. This analysis revealed nine discrete modules (Additional file 1: Figure S1) whose genes clearly correlate with a metabolic or specific function (Figure 2), as reported for *E. coli* and *M. tuberculosis*[6,11,12].

### Granularity of the detected modules

In this work we identified modules using the hierarchical clustering method originally proposed for protein networks by Rives and Galitski [3] and applied for the first time to regulatory networks by Resendis-Antonio et al. [6]. To evaluate the identified modules, we compared our results to three alternative methods for modules detection: Girvan-Newman [2], Rosvall-Bergstrom [13], and the natural decomposition approach (NDA) [14]. In general terms, the three methods re-captured the identified modules obtained originally with the hierarchical clustering method, although showed different granularity (see Additional file 2: Table S1).

The identified by the Girvan-Newman algorithm showed the highest similarity with the ones identified with the hierarchical clustering. There were only two differences: 1) PhoP was clustered into a different module, and 2) module 8 was disaggregated into two submodules. The Rosvall-Bergstrom algorithm also clustered PhoP into a different module in addition of a more disaggregated modules. Rosvall-Bergstrom found that some elements of the modules 6, 7, 8 and 9 could be disaggregated into 2, 2, 4 and 3 submodules, respectively.

Interestingly, module 6 identified by Rosvall-Bergstrom is the more transverse module, which is dispersed over three modules identified by hierarchical clustering (modules 5, 6 and 7). The NDA mathematically identifies global TFs and remove them from the network. As a consequence of this step, global TFs are not clustered into any module. This allowed the finest disaggregation into submodules of the four methods. No transverse modules were identified with this method. The transverse module 6 identified by Rosvall-Bergstrom was identified by the NDA as two modules with no functional correlation (13 and 54) and two global TFs. Despite this finer granularity, the physiological functions annotated for each module identified in this work highly correlated with the corresponding functions for the submodules identified by the NDA. We observed that the modules identified by any method are mainly subsets or supersets of the modules identified by other method. These results highlight the relevance of taking into account the previously reported *Matryoshka*-like organization of regulatory networks [14] by showing that while different methods are able to re-capture the identified modules, this is accomplished at different granular levels.

### The modules of the *B. subtilis* TRN clearly correlate with well-defined metabolic and physiological responses

To characterize the metabolic and physiological responses of each of the nine modules identified in the *B. subtilis* TRN, we performed an exhaustive literature search of the experimentally validated regulatory data for each response. The complete description of each module and its relationship with their regulated genes and other modules can be viewed in the Additional file 1.

#### ***Module 1 (M1)***

Groups the TFs TnrA, GlpR, and KipR, regulating genes involved in *Nitrogen assimilation function*s (Figure 2 and Additional file 1: Table S2). TnrA is required during nitrogen-limited growth and GlpR during growth with excess nitrogen [15]. TnrA regulates KipR, also detected in this module, whose main function is displayed during the sporulation cell fate [16] (more details are provided in the Additional file 1).

#### ***Module 2 (M2)***

Devoted to *Nucleotide synthesis* includes the PyrR TF, which regulates pyrimidine synthesis and metabolism by transcriptional attenuation [17], and PurR, which regulates genes involved in purine and pyrimidine synthesis and transport [18,19] (Figure 2 and Additional file 1: Table S2).

#### ***Module 3 (M3)***

Cluster the TFs CssR and HtrA, which are expressed under stressful conditions are regulate genes related to *Secretion*[20] (Figure 2) and (Additional file 1: Table S2).

#### ***Module 4 (M4)***

Entitle as *Degradative enzyme* module, is integrated by two TFs, TenA and TenI (Figure 2), that regulate the production of several extracellular enzymes [21].

#### ***Module 5 (M5)***

**Figure 3:**
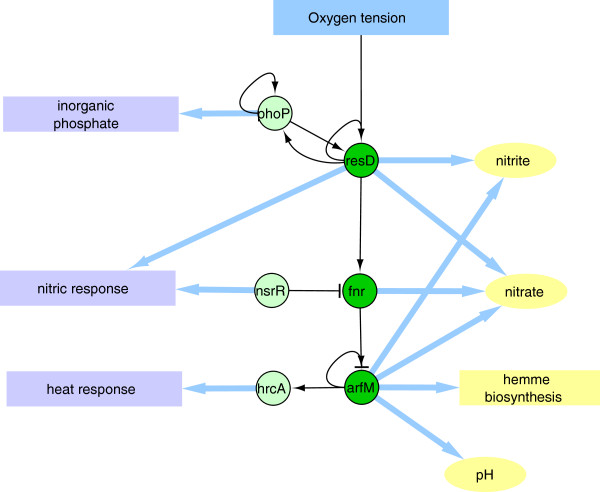
**Respiratory functions.** Regulatory cascade associated with the gene induction under low oxygen conditions (dark green circles). As described in the text, other TFs (light green circles) were also clustered in this module, for which functions in the adaptation to respiratory stress have been described.

Or *Respiration* module includes all the TFs that are required for switching between aerobic and anaerobic growth (see Additional file 1: Table S2). The TFs belonging to this module are ArfM, HrcA, FNR, NsrR, ResD, and PhoP, which are highly inter-regulated in a hierarchical order (see Figures 2 and 3). The complex regulation of this module correlates with the fact that *B. subtilis* grows either by fermentation or anaerobically, using nitrate or nitrite as terminal electron acceptors [22].

#### ***Module 6 (M6)***

Devoted to *Carbon metabolism,* groups the TFs CcpA (Figure 2 and Additional file 1: Figure S1). CcpA is the master regulator of sugar operons (see Additional file 1: Table S2 and a detail description in the Additional file 1), which regulates almost all the TFs in this module except for CcpB. GntR a TF that is responsible for gluconate catabolism regulation [23] is an example of this. ExuR involved in hexuronate assimilation, is regulated by CcpA and σE, which are located in the *CMCS module*. Other proteins also regulated by CcpA, are AcoR a regulatory protein that is expressed when *B. subtilis* is in the exponential growth phase and excretes diverse organic compounds, such as acetoin, TreR that coordinates the expression of different kind of genes in response to trehalose availability (Additional file 1: Table S2), GlvR involved in the maltose utilization [24], FadR involved in the fatty acid β-oxidation cycle and CcpC necessary for the catabolic repression of genes that are involved in the Krebs cycle [25,26].

#### ***Module 7 (M7)***

Cluster the TFs controlling the *Cascade of the mother cell sporulating (CMCS)*, the genes encoding the TFs of this cascade are expressed hierarchical in the following order *sigE → spoIIID* and *gerR → sigK → gerE* and *yfhP*[27,28] (see Figure 2, Additional file 1: Table S2 and annotations in Additional file 1)*.*

#### ***Module 8 (M8)***

**Figure 4:**
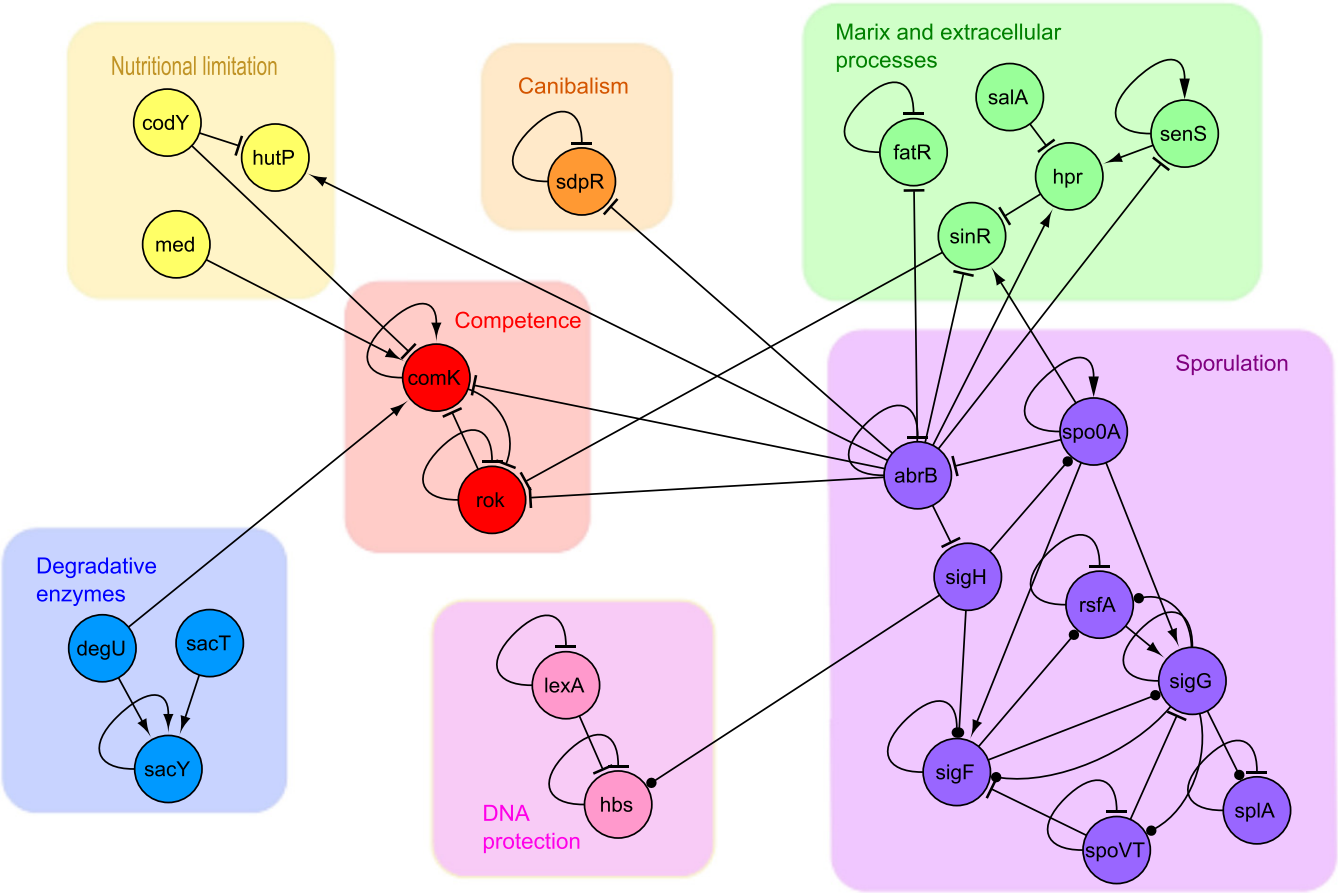
***B. subtilis *presents different cell fates.** TFs clustered in the *cell differentiation* module are devoted to different phenotypical subpopulations. Each color box emphasizes the group of TFs belonging to each related function.

Groups TFs involved in *Cell differentiation*, involving four master regulators: AbrB, DegU, ComK, and Spo0A [29], that coordinate in conjunction with other regulators the following well-defined cellular responses and fates: *sporulation*, *competence*, *DNA protection*, *matrix and extracellular protein biogenesis*, *cannibalism*, *degradative enzyme synthesis*, *and nutritional limitation response* (Figure 4 and Additional file 1: Table S3), that were clustered together in this module. In the Additional file 1, we discuss the direct and indirect influence of the master transcriptional regulators, on various differentiation processes and stress responses and its relationship with other TFs coordinating the above mention fates and cellular responses.

#### ***Module 9 (M9)***

In addition to cell differentiation, *B. subtilis* has other methods to face adverse growth conditions. The genes involved in these activities are regulated by different σ factors and TFs clustered in the *General stress module.* The σB regulon is one of the alternative responses, and it is activated to protect the vegetative cell during starvation or physical stress [30]. The stress responses includes TFs such as CtsR, BmrR, YtlI, CymR, PerR, YodB, LytR, YvrH.and Spx, and the σ factor YlaC, σM, σW, and σX (see Figure 2), which specific function are described in the Additional file 1.

### Paralogy; an evolutionary force modifying the TRN of B. subtilis

In a previous study, we performed an exhaustive review of paralogous gene regulation in *E. coli* and *B. subtilis* based on published information [31]. In this work, we identified the paralogous TFs in the constructed TRN and briefly discussed the implications of the distribution of the TFs inside and between modules.

**Figure 5:**
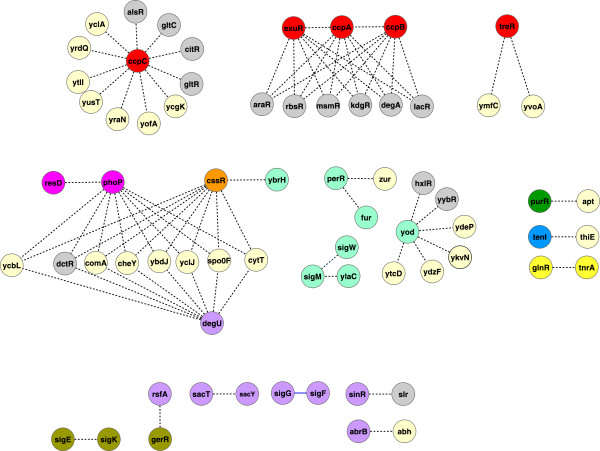
**Paralogous transcription factors not belonging to a module.** In red are the paralogous TFs belonging to the *carbon metabolism* module. Paralogous TFs associated with the *general stress* response module are indicated in light green. TFs involved in *nucleotide biosynthesis* are shown in dark green. TFs devoted to the *respiration* module are shown in pink. TFs related to the *secretion stress* module are shown in orange. Paralogous regulatory proteins clustered in the *cell differentiation* module are shown in purple. TFs regulating *nitrogen metabolism* are shown in yellow. Paralogous TFs clustered in the *mother cell* module are shown in brown. TFs representing *degradative enzymes* are shown in blue*.* A group of regulatory genes that were not clustered in a module (grey) but are paralogous to a TF belonging to a module is also shown. A group of structural genes that are related to paralogous TFs are shown in light yellow. The line represents the paralogous relationship between structural and/or regulatory genes.

In our previous study, we found that TnrA and GlpR located in M1, are paralogous proteins (Figure 5) [31] that belong to the MerR family [32], and interestingly, their DNA-binding sites have the same consensus sequence [33]. A large fraction of neighboring TF binding sites have been formed by local duplications of a common sequence and might diverge as a consequence of point mutations [34]; further, these sites may have been selected for specific environmental conditions, as suggested by Singh and Hannenhalli [35]. Additional examples of interchangeable DNA-binding sites have been observed in other families of *E. coli* regulatory proteins, such as CRP and FNR [36].

As in previous works [31,35,37,38], we observed that some duplication events does not give as a result two TFs. An example of this, is PurR cluster in M2, whose paralogous copy is the protein Apt, an adenine phosphoribosyltransferase [39] (Figure 5) that participates in nucleotide synthesis and is an enzyme rather than a TF. Both proteins have a PRT motif that is involved in the binding of the inducer phosphoribosylpyrophosphate. In PurR, this domain is fused to a winged-helix-turn-helix domain that is present in other DNA-binding proteins, while in Apt, the PRT domain presents catalytic activity [40]. This module, is a clear example of how the diversity of paralogous proteins and gene functions, might increase the genetic and metabolic robustness of a network. We observed something similar in M3, where the TFs CssR and HtrA are not homologous. Instead, CssR have as paralogous copy the TF YvrH, positioned in M9 that group TFs related with the *General stress* response (Figure 5) [31], As CssR, YvrH is a response regulator of a two-component system (YvrG-YvrH), but differently to CssR the regulatory function of this TF participates in the control of the homeostasis of *B. subtilis* at the cell surface level [41]. This example, illustrates that paralogous TFs do not always regulate genes that are related to functional or metabolic processing but can be part of different regulatory modules. Similar results on the plasticity and robustness of the regulatory networks of *E. coli* and *S. cerevisiae* have been described by Babu and Teichmann [38] and Conant and Wagner [42].

In M4 the only members of the module, TenA and TenI are not homologous; nevertheless, TenI has a paralogous copy with catalytic activity: the ThiE thiamine-phosphate pyrophosphorylase enzyme (Figure 5) [43]. Similar examples were found in M2 and M3, related with *Nucleotide synthesis* and *secretion stress*, this confirms that paralogous proteins with different functions might increase the plasticity and robustness of the *B. subtilis* regulatory and metabolic network.

In M5 we observed that the TFs ResD and PhoP, based on their sequence similarity, evolved from a common ancestor [44] and form part of the IIIA group of two-component systems (Figure 5). These similarity is worth noting not only because these paralogous TFs coordinate the expression of genes that are involved in the phosphate uptake, but also because they are part of a paralogous set of two-component system regulatory proteins, ResD/ResE and PhoP/PhoR, where ResE and PhoR are membrane-bound histidine kinases that sense the extracellular phosphate concentration [45]. Whitworth and Cock previously postulate, that genes regulated by two-component systems might allow rapid and robust responses to short-term changes in the environment [46].

CcpB is a paralogous copy of CcpA, the main TF in the *Carbon metabolism module* M6. These TFs share 30% sequence similarity, and as in the case of CcpA, the down-regulating activity of CcpB depends on the phosphorylated state of HPr. In parallel with CcpA, CcpB regulates the *gntR* regulatory gene and the *gnt* and *xyl* operons, which are involved in the metabolism of gluconate and xylose, respectively [32]. ExuR is another CcpA-paralogous copy in the *Carbon metabolism* module, the activity of which is down-regulated by the phosphorylated state of HPr (See Figure 5). In our previous work [31], we described many other CcpA paralogs in *B. subtilis* that are different from CcpB and ExuR. These TFs were not included in our network because no regulatory interaction different from σA, has been reported for any of them in the DBTBS database. A similar situation also exists for the paralogous copies of the TFs CcpC and TreR.

### Master regulators govern sporulation and cross-talk with other modules

A topological motif is defined as a statistically over-represented pattern of interconnected nodes and links (subgraphs) in a complex biological network [47]. Recent evidence suggests that motifs in regulatory networks could be a by-product resulting from network organization and evolution [48-51]. Two principal network motifs have been found in TRNs: the feed forward motif (FF) and the bi-fan motif (BF) [7]. FFs are three-network motifs that comprise two regulatory genes and one target gene (A → B, B → C, A → C), and BFs involve two regulatory genes and two target genes (A → C, A → D, B → C, B → D). Some studies have suggested that FFs play important organizational [49,52] and dynamical [53,54] roles that could explain why they have been selected in TRNs, whereas other studies have shown that the overabundance of BFs does not correlate with any specific functional role [55]. Hence, we only focus our attention in this work on FFs that perform various functional roles, including noise filtering, fine tuning of expression timing, response acceleration, and pulse generation, all of which are well described in the context of sporulation in *B. subtilis*[56].

We used the *CMCS* to exemplify the relevance of FFs and to highlight the possibility that FF regulatory genes perform cross-talk regulation between this module and others. We searched for the entire set of three-node network motifs in the *B. subtilis* TRN, as described in the methods section. Based on this analysis, two motifs were identified: the FF and an alternative version consisting of a two-node feedback circuit between the regulatory nodes. The latter, herein called the complex feed forward motif (CFF), has also been identified in the *E. coli* TRN, which highlights the importance of feedback circuits for TRN organization [47,49]. At the global scale, 89% of the FFs and 100% of the CFFs in the *B. subtilis* TRN are embedded within specific modules, while the remaining FFs enable, at the level of regulatory genes, cross-talk between modules (Table 1).

**Table 1:** **Motif distribution in the *Cascade of the mother cell sporulating *** **module in the *B. subtilis *** **TRN**

**Master regulator (module)**	**Local regulator (module)**	**Number of target genes**
SigE (sporulation)	SigK (sporulation)	11
SigE (sporulation)	SpoIIID (sporulation)	48
SigE (sporulation)	YlbO (sporulation)	11
SigE (sporulation)	ExuR (carbon metabolism)	7
SigE (sporulation)	PhoP (respiration)	4
SpoIIID (sporulation)	GerE (sporulation)	4
SpoIIID (sporulation)	SigK (sporulation)	7

The two regulatory genes involved in a FF could be classified into master and local regulators. The master regulator governs the expression of the local regulator and the target gene, while the local regulator only governs the expression of the target gene (Table 1). Due to the hierarchical nature of the *B. subtilis* TRN, a gene that is considered to be a master regulator in a given FF could appear as a local regulator in another FF [14,49,57]. However, by considering the number of FFs in which a TF is a master or local regulator, it is possible to infer its role in the entire TFN.

### Applying these criteria to the FFs in the *CMCS* module, we found that SigE and SpoIIID could be classified as master regulators

The gene targets of SpoIIID regulation are also targets of GerE or SigK regulation. In contrast, the gene targets of SigE regulation are also commonly targets of SigK, SpoIIID, or GerR TFs. In some cases, SigE (*CMCS* module), ExuR (carbon metabolism module), and PhoP (respiration module) regulate the same set of genes, thus enabling regulatory cross-talk between these three modules.

**Figure 6:**
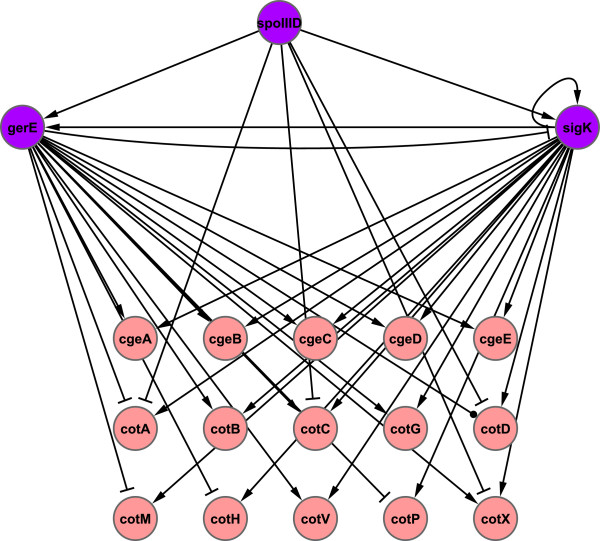
**Motif over-representation in the *Cascade of the mother cell sporulating *module.** GerE and SigK TFs are involved in a two-node feedback circuit that coregulates a large set of target genes.

A similar analysis of the CFFs indicates that GerE and SigK, which are both involved in a two-node feedback loop, coregulate a large set of common target genes (Figure 6). As discussed in a recent paper published by our group [14], the feedback loops, are not over represented structures but no for that less important. As in the presented circuit formed by the TFs GerE and SigK for which an experimental study has provided evidence showing that this FBL plays a key role in enhancing the robustness of the mother cell network and optimizing the expression of target genes [58].

### The role of σ factors in the TRN

In our initial attempt, we performed an analysis of the *B. subtilis* TRN considering only TFs in the absence of any σ factor (data not shown) and found that the resulting network was biologically meaningless because it was decomposed into a very large number of small modules, many of which shared the same function or metabolic process. For example, we found multiple *Respiratory*, *Sporulation*, and *Carbon compound* modules. In addition, we found that the number of TFs in this TRN was reduced when the σ factors were not considered because they were the only connections of many TFs to the regulatory network. In some cases, the loss of TFs from the TRN led to an absence of specific functional descriptions in the resulting modules. In contrast, the inclusion of σ factors in the *B. subtilis* TRN generated cohesive modules associated with well-defined physiological functions and cell processes that are characteristic of this model organism. Furthermore, the 11 *B. subtilis* σ factors included in the analysis were also grouped into functional and characteristic modules (see Figure 2). For example, the presence of σL in the *Carbon metabolism* module established regulatory links with TFs that regulate genes involved in the metabolism of fructose, levanase, arginine, acetoin, isoleucine, leucine, and valine. In addition, σK and σE participate in a regulatory cascade that is required for sporulation and were clustered in the *CMCS* module, while σH, σF, and σG were clustered in the *Cell differentiation* module and are responsible for different stages of the spore formation: initiation of sporulation, early spore formation, and late spore formation, respectively. It is also important to observe that σE and σF were assigned to different modules and are instrumental in preparing for cell-specific programs after the septum formation. The remaining five σ factors, σB, σM, σW, σX, and YlaC, were organized into one module whose function is associated with general stress response. The σB factor is considered to be the master regulator of the stress response in Gram-positive bacteria, while the other four factors belong to the extracytoplasmic function σ factor family, which is characterized by a response to various stress factors [59]. The aforementioned examples suggest that the inclusion of σ factors in the construction of the TRN provides relevant information regarding their important regulatory roles in the metabolic and cellular processes that take place in *B. subtilis.*

### Different functions in one module

A meticulous analysis of the regulatory roles of the TFs showed that each module typically has one function or is related to a specific metabolic process. However, in some modules where more than one function was identified, meaningful biological relationships between these functions were discovered. In this section, we discuss these cases.

*The respiration module* cluster has six TFs, ArfM, Fnr, NsrR, ResD, PhoP and HrcA. The first four TFs are part of a regulatory cascade that is triggered in response to changes in oxygen availability in the environment and regulate the expression of genes required for the cell adaptation from aerobic to anaerobic environments, and *vice versa*. PhoP is a TF whose expression is induced when decreasing the presence of phosphate in the medium and shares common regulated gene targets with ResD, that are involved in the first steps of the respiratory process. This concurrent regulation reflects the dependent relationship of the bacterial respiratory process with the phosphate availability in the media. Furthermore, transcription induced by ResD under limited phosphate conditions provides essential components for the transport of electrons, required for the assimilation of inorganic phosphate into ATP [45].

The sixth TF clustered in the *respiration module* is HrcA that participates in heat-shock stress response regulation [60] and is encoded in the *lepA-hemN-hrcA-grpE-dnaKJ-yqeTUV* operon. The heat-shock-related genes of this operon include the *grpE-*, *dnaK-*, and *dnaJ*-encoding chaperons*.* This operon also encodes for genes required in the respiratory process, such as the GTP-binding protein LepA and the oxygen-independent coproporphyrinogen III oxidase HemN, which participates in heme biosynthesis and anaerobic respiratory energy metabolism [61,62]. The *yqeT* gene codes for a protein that is homologous to the L11 methyltransferase ribosomal protein, while *yqeU* and *yqeV* code for proteins with unknown function [61,62]. Transcription of the *lepA-hemN-hrcA-grpE-dnaKJ-yqeTUV* operon is controlled by at least four promoters that depend on σA, three terminators, and ArfM and is autoregulated by HrcA [62,63]. One start site that responds to aerobic/anaerobic conditions is located 37 bp upstream of the *lepA* translational start codon [63]. The remaining sites are located downstream of *hemN* and respond to heat-shock stress [64]. The means by which ArfM controls the expression of this operon in anaerobic conditions remains unclear, although a cascade of events in which ResD favors the expression of FNR, a mediator of the anaerobic induction of ArfM, may be responsible for the activation of the *lepA-hemN-hrcA-grpE-dnaKJ-yqeTUV* operon [65]. However, more experiments need to be performed to confirm this hypothesis.

The *Cell differentiation* module is another example where different but related functions are observed together. This module contains the most TFs, and although most have a direct implication in the phenotype expressed in each cell fate, there are some genes whose presence warrants further discussion. Two of them code for the LexA and Hbs TFs, which are associated with the care, protection, organization, and structuration of DNA. Hbs has an important effect on gene expression and growth; thus, it is required not only during sporulation, but also during vegetative growth [66]. Mutants of this gene show reduced sporulation efficiency [66]. In contrast, LexA is a master regulator of genes that is involved in DNA damage and the SOS response, and it has a role in coordinating the initiation of sporulation when the cell is exposed to DNA damage. Finally, HutP is directly involved in the use of alternative carbon and nitrogen sources [67], but was clustered in the *Cell differentiation* module because it is regulated by the master regulator AbrB and the CodY TF, which regulate gene expression during the competence state. Additionally, HutP is regulated by CcpA, the master TF for carbon metabolism. Sporulation is a critical cell fate that allows *B. subtilis* to adopt a resistant structure, thereby allowing its survival in extreme, unfavorable conditions. The decision to begin the sporulation process is critical because once the process is started, it will continue until it has been completed, committing these cells to a latent state of viability. For this reason, the transcription regulation of genes involved in sporulation must be concurrently regulated by TFs like HutP and CcpA in response to the availability of nitrogen and carbon sources.

One cluster that certainly represents a module with more than one function is the *General stress response* module. This module is coordinated by the master regulator σB, which controls the expression of more than 150 genes that are involved in *B. subtilis* adaptation to different types of stresses and starvation stimuli typical of its natural ecosystems [68]. A cascade of regulatory events induces the activity of σB, which is a consequence of a switch mechanism that phosphorylates the proteins RbsV and RvsW as the final intermediates of the cascade. RvsW captures σB in a stable complex that prevents the union of σB with RNApol, a condition prevailing in exponential growth. This condition is reverted when *B. subtilis* is exposed to stress, for which two modes of action have been described [68]. The first response is induced by environmental stresses such as heat-shock, salt, acid, ethanol, Mn2+, and blue light [68]. The second response is induced by energy depletion and requires the detection of glucose, oxygen, and phosphate starvation and exposure to agents such as NO, azide, CCCP, and mycophenolic acid [68]. As a consequence of these stresses, a portion of the cells commits to sporulation, but the rest rely on alternative survival strategies. One such strategy is provided by σB or the structural protein RelA and consists of coordinating a stringent response that can “lead to a vegetative dormancy characterized by drastically reduced anabolic reactions and a prospective protection by a multiple stress resistance machine directed mainly by different sets of TF,” as described by Hecker and colleagues [68].

### Functions present in more than one module

In our network analysis, we were able to define regulatory modules with specific metabolic or cellular function; nevertheless, we found two cases in which one function was redundantly regulated by TFs belonging to different modules of the network. These cases were associated with the regulation of heat-shock proteins and the regulation of degradative enzymes synthesis. These cases are discussed below.

One hardship that bacteria must face is growth adaptation to different temperature changes, for which they have developed diverse programs of gene regulation. The response to heat-shock stress is one of the best-known systems and involves the so-called heat-shock proteins (HSPs). In *B. subtilis*, more than 200 genes are induced in response to a heat shock. These genes are regulated through different mechanisms that provide a method of classification. Based on their regulatory elements, HSPs have been classified into six main classes or regulons [69]. Although all of the genes of these regulons are classified as being involved in heat shock, many of them also respond to a variety of other stress stimuli; therefore, their corresponding TFs are included in more than one module. For example, class III is regulated in response to heat-shock stress and oxidative stress by CtsR, which belongs to the *General stress module*[70], while class V is regulated in response to heat-shock and secretion stress by the CssRS system, which belongs to the *Secretion* module.

In contrast, Class II and Class III heat-shock proteins were grouped into the *General stress module* and are regulated by σB in response to heat-shock and a diversity of other stresses. The activity of σB is controlled by a complex signal transduction network that includes at least seven other gene products encoded by the *sigB* operon [71]. This complex regulation might allow the efficient adaptation of *B. subtilis* to a broad diversity of stresses and starvation in non-sporulating conditions [72] (Figure 2). Finally, Class V HSPs were grouped in the *Secretion stress* module that is regulated by the two-component system CssR-CssS, with a possible dual role of CssRS in the heat-shock and secretion stress responses [73]. Our above description of the heat-shock response in *B. subtilis* demonstrates that modular analysis is a tool that can be used to understand potential relationships among the different regulons of the heat-shock stress proteins. Furthermore, this type of analysis could be expanded by comparing the regulatory networks of different organisms. For example, in the proteobacteria *E. coli,* only one heat-shock regulator, the σ32 has been described at the transcription level, while in *B. subtilis* and others Gram-positive organisms, several mechanisms regulate the heat-shock response. In this regard, the complex regulation of *B. subtilis* may allow this organism to tolerate the extensive stresses that are present in the different and changing environments where it grows [69].

The presence of the same function in different modules was also observed in the transcription regulation of degradative enzymes, which clustered in the *Degradative enzymes* and *Cell differentiation* modules. In the first module, the transcription of the genes coding for degradative enzymes are regulated by TenA and TenI, while genes in the second module are regulated by SenS and DegU, which also regulate the expression of genes responsible for biofilm formation. TenA and TenI seem to be unessential for the production of degradative enzymes in *B. subtilis*. These TFs might compensate for the function of SenS and DegU in the case of mutation. In this and other instances, redundancy in regulatory elements might increase the robustness of biological systems [74,75].

Sporulation is another cellular process that appears in more than one module. In our analysis, we found that different sets of regulatory elements were associated with this process in the *CMCS cell* and *Cell differentiation* modules. Sporulation is primarily controlled by Spo0A and σH, which are both involved in the initiation of this process, and by genes that are expressed in the regulatory cascade that occurs in the forespore. The separation of both groups of genes into two modules originates from the compartmentalization of the mother cell and forespore gene expression during endospore formation, which is triggered by the phosphorylated form of Spo0A.

## Conclusions

The work here presented reviewed the TRN of *B. subtilis* by an exhaustive manual functional annotation of the identified regulatory modules and motif distributions.

In good agreement with previous works, we found that the *B. subtilis* regulatory network displays the typical characteristics of a scale-free network with modular and hierarchical organization. The determination of these properties allowed us to classify the TFs into nine discrete modules that are highly connected in an intra-modular fashion and show a hierarchically organized inter-modular structure (See Figure 2). The detailed literature analysis demonstrated that each module was associated with well-defined physiological functions. Although the modules were not entirely homogeneous in some cases, their components respond to common conditions or stimuli and were mainly regulated by global or pleiotropic TFs as describe previously for *Saccharomyces cerevisiae* and *Escherichia coli*[76]. In addition to the TFs, we showed that an important element of our analysis was the inclusion of the σ factors. This addition allows the clustering of TFs into larger and more biologically meaningful modules than those obtained in our previous study in *E. coli*, where the σ factors were not considered [6]. The advantages mentioned above seem to be more evident in organisms with many σ factors, such as *B. subtilis*, which has 16 σ factors [1], compared with organisms with fewer σ factors, such as *E. coli*, which has only seven σ factors in its regulatory network [77]. In the particular case of *B. subtilis,* important cell fates, such as sporulation and the general stress response, depend on cascades of σ factors. These regulatory relationships were observed in our analysis of regulatory modules, in which some σ factors can play the role of pleiotropic regulators [14], governing different cell fates and cross-talking; a classification that can be done though the analysis of motifs like FFs, and FBLs and the previously proposed intermodular genes [14]. In good agreement with previous studies on the evolution of cellular networks by duplication events [31,35,37,38], we identified paralogous TFs playing important roles in the TRN of *B. subtilis*. In summary, the analytical methodology utilized in our study provides an excellent approach to integrate and understand the complex regulatory network of *B. subtilis*, a network that modulates the expression of the different mechanisms of adaptation and cell differentiation that this bacterium has evolved in response to the environmental changes that it must constantly face.

## Methods

### Network construction

From DBTBS version 2010 [1], we extracted all the regulatory interactions between TFs, σ factors, and target genes reported in this database. We selected regulatory interactions with strong evidence, using as a parameter the strong and weak evidence classification performed in the RegulonDB database [77]. The full TRN of *B. subitilis* and the TFs and σ factors can be consulted and download from the Additional files 3 and 4 respectively and or the address provided by the journal.

### Statistical analysis of the regulatory networks

The connectivity (P(k)), and the clustering coefficient (C(k)) distributions of the *B. subtilis* network were obtained as described in Resendis et al. [6]. Our analysis shows that the transcriptional regulatory network of *B. subtilis* follows a scale-free distribution with hierarchical modularity. We also compute (see the methods section) the incoming (*Pin*) and outcoming (*Pout*) degree distributions for three bacteria (*B. subtilis*[14], *E. coli*[78] and *M. tuberculosis*[79]) and one eukaryote (*S. cerevisiae*[80]) to visualize and understand generic characteristics of the regulatory network topology. Additionally, we computed the coefficient of determination (R^2) for each regression as an indicator of the goodness of fit of the power-law model, and then compared each of them against the R^2 for a corresponding exponential fit.

In a posterior step, we extracted a sub-network considering only the regulatory interactions of all known *B. subtilis* TFs and σ factors. Over this sub-network, we calculated the shortest path length between every pair of genes (d_ij_ is the shortest path length between gene i and gene j). Next, we calculated the association function (1/d_ij_^2^) of these shortest path lengths [3,6]. This function gives a measure of the closeness among genes by amplifying close relationships and minimizing remote distances. The data were used as input to perform a hierarchical agglomerative average linkage clustering using the programs Cluster 3 [81] and TreeView [56] for cluster visualization.

### Modules homogeneity

With the aim to corroborate the homogeneity of the modules obtained by the hierarchical clustering method, we performed three extra clustering algorithms: the Girvan-Newman algorithm [2], the Rosvall-Bergstrom algorithm [13] and the Natural decomposition approach [14].

### Manual annotation of identified modules

We annotated each module as follows. First, we compiled a list of cellular processes in which the genes composing each module were involved. We obtained this information from the DBTBS (version 2010) database [1] and a review of pertinent literature. Then, taking into account the regulatory and physiological context, this information was analyzed using expert biological knowledge to make human inferences about the physiological function of each module.

### Motif identification

We searched for the entire set of three-node network motifs in the *B. subtilis* TRN by using the *mfinder* program [47]. To determine the statistical over-representation of three-node subgraphs, a switching algorithm was used to generate 1,000 random networks that conserved the number of nodes, links, and the degree sequence of the real network. All three-node subgraphs showing a *p*-value < 0.01 were selected as network motifs.

### Availability of supporting data

The data sets and the following information supporting the results of this article are included within a Additional files 1 and 2. An address with down load files of the full TRN of B*. subtilis* and the TFs and σ factors TRN of *B. subtilis* can be download from Additional files 3 and 4 respectively.

## Abbreviations

TF: DNA-binding transcription factor; TRN: Transcriptional regulatory network; CMCS: Cascade of the mother cell sporulating module; FF: Feed forward motif; CFF: Complex feed forward motif; CCR: Catabolite repression; NDA: Natural decomposition approach; M: Module; Pin: Incoming degree distribution; Pout: Outcoming degree distribution; CCDF: Cumulative complementary distribution.

## Competing interests

The authors declare that they have no competing interests.

## Authors’ contributions

JAFG reconstructed the TRN, performed topological, module identification and motifs analyses and contribute to writing. AMMC designed the study, collected the data and analyzed the results. EM was involved in draft and revising the manuscript. MMN contributed with the paralogous assignations. EPR was involved in revising the manuscript. RMGR designed the study, collected and analyzed the data, and wrote the paper. All authors read and approved the final manuscript.

## Supplementary Material

Additional file 1 **Lessons from the modular organization of the transcriptional regulatory network of *Bacillus subtilis.***
Click here for file

Additional file 2: Table S1 Comparison between different clustering methods. Click here for file

Additional file 3 B. subtilis full TRN. Click here for file

Additional file 4 Transciptional regulatory network of TFs of Bacillus subtilis. Click here for file
