# Supplementary material for: Lessons from the modular organization of the transcriptional regulatory network of Bacillus subtilis
Source: BMC Syst Biol. 2013 Nov 16;7:127. doi: 10.1186/1752-0509-7-127 (PMC4225672; doi:10.1186/1752-0509-7-127)
Supplement: Additional file 1 — Lessons from the modular organization of the transcriptional regulatory network of Bacillus subtilis. [file 1752-0509-7-127-S1.docx]

Supplementary material

**Lessons from the modular organization of the transcriptional regulatory network of *Bacillus subtilis*.**

Julio A. Freyre-González^1^, Alejandra M. Manjarrez-Casas^2^, Enrique Merino^2^, Mario Martinez-Nuñez^2^, Ernesto Perez-Rueda^3^, and Rosa-María Gutiérrez-Ríos^2*^.

^1^ Programa de Genómica Evolutiva, Centro de Ciencias Genómicas, Universidad Nacional Autónoma de México, Av. Universidad s/n,Col. Chamilpa, 62210, Cuernavaca, Morelos, México. ^2^ Departamentos de Microbiología Molecular and ^3^ Ingeniería Celular y Biocatálisis. Instituto de Biotecnología, Universidad Nacional Autónoma de México, Apdo. Postal 510-3, Cuernavaca, Morelos 62250, México.

*Corresponding author: Rosa-María Gutiérrez-Ríos (rmaria@ibt.unam.mx)

**INDEX.**

1. **Figure S1. A heat map reflecting the modular organization of the TRN of TFs of *B. subtilis,* using the hierarchical clustering method**
2. **The *Bacillus subtilis* TRN.**
3. **Table S2. TF and its regulatory function**
4. **A full description of the modules of the *B. subtilis* TRN** **and its** **correlation with well-defined metabolic and physiological responses.**
5. **Figure S1. Hierarchical clustering.**

**
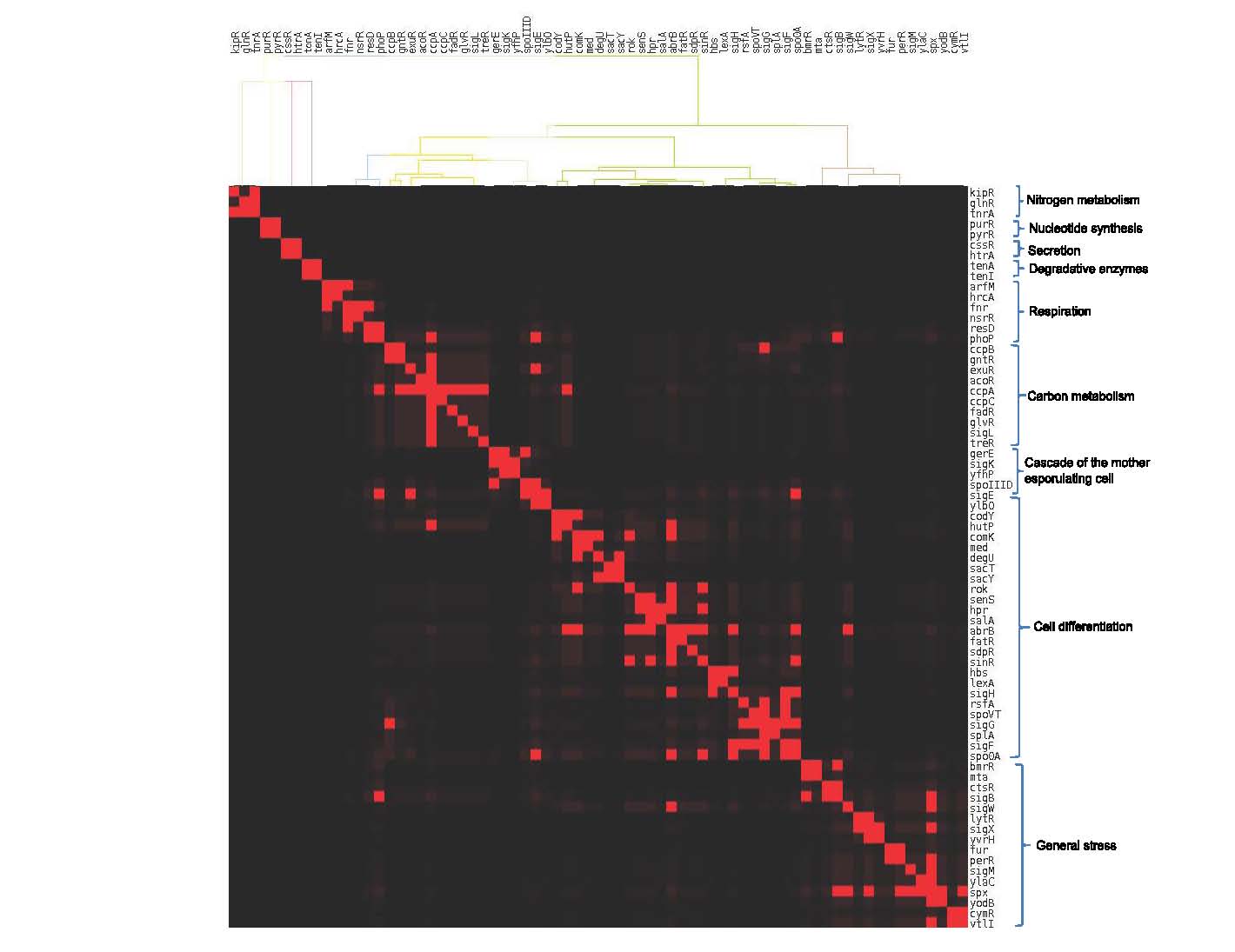
**

1. **The *Bacillus subtilis* TRN.**

**Appendix 1. Full TRN.**

The full *B. subtilis* TRN is available for download as ***Supporting Table***, in a format that facilitates network filtering based on the source (and reliability) of gene regulatory interactions. The meaning of the columns in this file is the following:

Column 1: Regulatory gene, traditional name;

Column 2: Target gene, traditional name;

Column 3: Regulatory function over target gene

Column 4: Target gene, GeneBank ID;

Column 5: Target gene, Locus Tag;

<http://www.ibt.unam.mx/biocomputo/Full_TRN_Bsubtilis.txt>

This address will change for one provided by the journal

**Appendix 2. Trasncription and sigma factor TRN.**

As the full TRN, the TF TRN of *B. subtilis* is available for download as ***Supporting Table***, in a format that facilitates network filtering based on the source (and reliability) of gene regulatory interactions. The meaning of the columns in this file is the following:

Column 1: Regulatory gene, traditional name or names.

Column 2: Target gene, traditional name or names.

<http://www.ibt.unam.mx/biocomputo/TRN_transcription_sigma_factors_Bsubtilis.txt>

This address will change for one provided by the journal

**The modules of the *B. subtilis* TRN** **clearly** **correlate with well-defined metabolic and physiological responses.**

We characterize the metabolic and physiological responses of each of the nine modules identified in the *B. subtilis* TRN, and performed an exhaustive literature search of the experimentally validated regulatory data for each response. In the following sections, we describe and discuss the most striking findings.

**Nitrogen metabolism module 1.** This was the first module identified in our study and contains three TFs involved in nitrogen assimilation functions (Table 3 Supplemetary material): TnrA, GlpR [1], and KipR (Figure 2). TnrA is a global regulator that activates and/or represses genes such as *nrgAB*, *nasABCDE*, *gabP*, and *glnRA* [2] during nitrogen-limited growth. During growth with excess nitrogen, GlpR is a repressor of genes that are associated with the synthesis of glutamine, glutamate, urease, and the *tnrA* gene, which encodes the global nitrogen regulator TnrA [2].

In a global context, sporulation begins under limited nitrogen conditions; nevertheless, neither TnrA nor GlnR is directly required for the sporulation process. However, TnrA regulates the expression of the operon *ycsFGI-kipIAR-ycsK,* which contains the KipR TF that is also included in this module. KipR, a member of the IclR family, regulates the expression of the *kipIA* genes. KipI functions as an inhibitor of the histidine kinase KinA, which is responsible for the phosphorylation of the sporulation protein Spo0F, and KipA counteracts the inhibitory effect of KipI [3].

**Nucleotide synthesis module 2.** Purines and pyrimidines are essential for energy transfer during metabolic reactions and are building blocks for constructing nucleic acids. Two TFs are included in this module (Table 3 supplementary material): PyrR, which regulates pyrimidine synthesis and metabolism by transcriptional attenuation [4], and PurR, which is a transcriptional repressor of the *pur* operon that codes for purine and pyrimidine synthesis and transport genes [5, 6]. PyrR is negatively regulated by both itself and PurR. Our study shows that these TFs and their regulated genes are only connected to the network through σA. These TFs, which have similar regulatory roles, were not generated by a duplication event, i.e., they are not paralogous [1].

**Secretion module 3.** *B. subtilis* is well known for its high capacity to secrete proteins into the environment. The main TFs of this cluster are CssR and HtrA, which are expressed under stressful conditions. CssR is the response regulator of the two-component system CssR-CssS that controls the gene expression of *htrA*, which encodes HtrA, a TF with serine protease activity that is required to degrade misfolded exported proteins that originated by heat-shock [7].

**Degradative enzyme module 4.** This module is integrated by two TFs, TenA and TenI, that regulate the production of several extracellular enzymes. These proteins are encoded together in the operon *tenAI-goxB-thiSGF-yjbV.* Although these two proteins are not essential for cell growth, their inactivation causes a delay in sporulation [8].

**Respiration module 5.** This module includes all the TFs that are required for switching between aerobic and anaerobic growth (see Table 3 supplementary material). The TFs belonging to this module are ArfM, HrcA, FNR, NsrR, ResD, and PhoP, which are highly inter-regulated in a hierarchical order (see Figures 2 and 4). The complex regulation of this module correlates with the fact that *B. subtilis* grows either by fermentation or anaerobically, using nitrate or nitrite as terminal electron acceptors [9]. The two-component system ResD-ResE participates in the first part of the regulatory cascade presented in Figure 4 and involves the following steps: under oxygen-limited growth, ResD induces the expression of *fnr*; the product of *fnr* (FNR) induces the expression of *arfM*; and ArfM modulates the expression of genes participating in anaerobic and fermentative growth [9], and the expression of HrcA, which is an important TF of the heat-shock response.

NsrR and PhoP are other TFs that are included in the *Respiration* module. NsrR regulates the expression of genes that respond to anaerobiosis when nitric oxide is present [10], while PhoP controls a regulon that responds to low levels of inorganic phosphate and is responsible for the phosphate uptake from complex substrates [11]. PhoP is positively autoregulated and is also regulated by ResD. The topology of this module reinforces a clear relationship between the respiratory process and the bacterial response under limited phosphate growth conditions [12].

**Carbon metabolism module 6.** As a heterotrophic organism, *B. subtilis* must obtain carbon from its medium, and glucose is the preferred source for this bacterium. However, in the absence of glucose, *B. subtilis* can assimilate alternative carbohydrate sources through different transport systems. These systems are precisely modulated by a complex regulatory system whose organization is reflected in this module (Table 3 supplementary material). The carbon metabolism assimilation of *B. subtilis* is regulated by a process known as catabolite repression (CCR) [13]. As showed in Figure 2 and Figure 1S, the highest connected TF of CCR is *Catabolite control protein A* or CcpA, which regulates almost all the TFs in this module and some other sugar operons The repressor activity of CcpA is triggered by the phosphorylated form of an intracellular sugar sensor protein called HPr [14]. Hpr is phosphorylated when glucose enters the cell; afterward, the CcpA-HPr complex binds to specific palindromic sequences known as *cre* sites that are located in the 5´ upstream regions of catabolic operons [15]. Within the *Carbon metabolism module*, CcpA regulates the expression of GntR, a TF that is responsible for gluconate catabolism regulation [16] and is subject to catabolite repression mediated by glycolytic intermediates. GntR is a transcriptional repressor of the *gntRKBZ* operon, and its down-regulating activity is antagonized by the presence of gluconate in the media [17]. Another TF cluster in this module is ExuR, which is regulated by CcpA and σE, which are located in the *CMCS module* (Figure 2). ExuR belongs to the *uxaC-yjmBCD-uxuA-yjmF-exuTR* operon, which is involved in hexuronate assimilation; the hexuronate operon can be induced with galacturonate as a sole carbon source. This polymer, also known as pectin galacturonic acid, is present in plant cell walls and, therefore, in soil, which is the most common growth medium for this bacterium [18].

CcpA regulates the expression of AcoR (Figure 2), a regulatory protein that is expressed when *B. subtilis* is in the exponential growth phase and excretes diverse organic compounds, such as acetoin. This molecule allows the bacteria to degrade large amounts of glucose without a notable acidification of the medium, and it can also be reused when other carbon sources have been exhausted [19]. The *acoABCL* operon encodes the enzyme complex acetoin dehydrogenase, which is responsible for the catabolism of acetoin in this Gram-positive bacteria [20], and the TF AcoR, which is responsible for the up-regulation of the operon [19]. In turn, the TF TreR, regulated by CcpA and itself, coordinates the expression of different kind of genes in response to trehalose availability. The utilization of this carbon source depends on the expression of the operon *trePAR*, and based on a bioinformatic analysis [21], the first gene *treP* exhibits high similarity to the IIBC enzymes of phosphotransferase systems from several organisms. *treP* may participate in the transport and phosphorylation of trehalose. The other gene, *treA*, encodes the intracellular-specific phospho-alpha-(1,1)-glucosidase (TreA), which hydrolyzes *in vitro* trehalose-6-phosphate and *para*nitrophenyl alpha-D-glucopyranoside [21]. As shown in Figure 2, the regulatory activity of CcpA is not restricted to the genes clustered in the *Carbon metabolism* module: it also acts as an intermodular regulator of other genes, such as *phoP* in the *Respiration module* and *hutP* in the *Cell differentiation module*.

Another TF involved in carbon metabolism that is clustered in this module is GlvR. This TF is part of the operon *glvA-glvR-glvC*, which is involved in the maltose utilization. Within this operon, *glvA* encodes a 6-phospho-alpha-glucosidase enzyme, while *glvC* encodes a PTS permease that participates in maltose uptake [22]. The *glvA-glvR-glvC* operon is positively regulated by GlvR and negatively repressed by the master carbon regulator CcpA [22].

Fatty acids are essential components of membranes and are important sources of metabolic energy in all organisms. In *B. subtilis*, the operons regulated by FadR (*lcfA-fadRB-etfBA*, *lcfB*, and *fadNAE*) [23], are involved in the fatty acid β-oxidation cycle and are subject to catabolic repression by CcpA. Under certain physiological conditions, such as the onset of sporulation, the *fadNAE (yusLKJ*) operon is induced, encoding enoyl-CoA hydratase, acetyl-CoA C-acyltransferase, and acyl-CoA dehydrogenase. This induction requires the SdpR protein involved in cannibalism and results in increased lipid oxidation and ATP production. An active metabolism and an increase in energy production may make cells more sensitive to antibiotics, which heightens their sensitivity to harmful factors by suppressing the antibiotic stress response and causing a delay in sporulation [24].

Besides controlling regulatory proteins, CcpA regulates the expression of σL, which is involved in regulating the catabolism of fructose [25], levanase [26], acetoine [19], and arginine [27]. This factor is also involved in the degradation of isoleucine, leucine, and valine [26, 28]. In addition, these carbon and amino acid metabolic pathways are connected with the cold-shock response through the regulation of σL [29]. This cold-shock response is a different stress compared to that produced by exposure to low temperatures, which is regulated by the general stress response regulator σB. The adaptation to a sudden low temperature shift induces the conversion of isoleucine to α-keto acids that are used as precursors for branched-chain fatty acid synthesis, which is required during the membrane adaptation to low temperatures [29].

Finally, the last TF regulated by CcpA and clustered in this module is CcpC. This TF is part of the *yhuJK-yzF-ykuL-ccpC* operon, which possesses three other genes of unknown function [30]. CcpC has been described as necessary for the catabolic repression of genes that are involved in the Krebs cycle [31, 32], mostly the genes that are associated with carbon metabolism, the sporulation process, and the uptake of divalent cations [33].

**Cascade of the mother cell sporulating (CMCS) module 7.** When nutrients are depleted from the medium, *B. subtilis* initiates a process called sporulation, the ultimate goal of which is to form a dormant and resistant structure: the endospore. One of the first changes in the cell is the formation of a septum that divides it into two asymmetric compartments: the stem cell and the forespore. Both compartments have different expression programs that derive from regulatory cascades initiated by different σ factors. Although both programs are clearly separated, both depend on each other in the TRN because they are connected by a signaling cascade [34]. In the mother cell, this cascade is hierarchical, and the genes are expressed in the following order: *sigE* 🡪 *spoIIID* and *gerR* 🡪 *sigK* 🡪 *gerE* and *yfhP* (Figure 2 and Table 3 supplementary material) [35, 36]*.* The first protein that regulates the sporulating function in the mother cell is σ factor E (σE), the transcription of which depends on the active phosphorylated form of Spo0A, which is located in the *Cell differentiation module* [37]. After asymmetric division, σE exerts its effect by initiating the transcription of diverse genes with multiple functions, such as engulfment of the forespore by the mother cell, prevention of a second asymmetric division in the mother cell, assembly of the spore coat, and maintenance of an adequate level of metabolic activity that allows the progression of the process of sporulation under nutrient-poor conditions [38, 39, 40]. In addition, σE regulates the transcription of *spoIIID* [41] and *gerR* [42], both TFs that were identified in this module. SpoIIID negatively regulates genes that are responsible for the cell morphological changes in the early stages of sporulation. SpoIIID also induces the transcription of genes that participate in the production of pro-proprotein σK, which takes its mature form as a result of the signaling network that connects the two compartments [35]. GerR represses fewer genes than SpoIIID; nevertheless, both TFs regulate the transcription of the *spoIIIA locus*, which encodes a set of eight proteins, SpoIIIAA through SpoIIIAH, that are necessary for continuing the process of sporulation after engulfment [35, 43]. Additionally, GerR regulates the *spoIIM* and *spoIIP* genes, which are involved in the elimination of peptidoglycan present in the septum dividing the cell. This elimination is a prerequisite for proper engulfment [44].

Several genes that are involved in the formation of the spore coat and spore maturation are controlled by σK [45]. This σ factor also regulates the transcription of *gerE* [46], as previously described. GerE partially controls the σK regulon, which is composed of genes that are activated at the end of the gene expression cascade within the mother cell compartment and are involved in the biogenesis of the endospore protein shell [35, 47]. Finally, this process allows the formation of the germ cell wall, a peptidoglycan layer between the innermost membrane surrounding the forespore and the cortex [36, 47]. The last TF described in this module is YfhP, the expression of which is regulated by σK; this TF seems to be a repressor of the *yfhQ- fabL-sspE* operon [48, 49]. YfhQ is a probable A/G specific adenine glycosylase; the FabL enzyme is involved in lipid metabolism; and *sspE* encodes a small protein of the spore that is soluble in acid [50].

Thus, the key regulators of the *CMCS* module are σE and σK. Other proteins primarily function to activate or repress genes in both regulons of these σ factors. These “on” and “off” gene states contribute to proper performance during the complex cell morphogenesis process that takes place during sporulation [35].

**Cell differentiation module 8.** Under stressful conditions, *B. subtilis* differentiates into distinct phenotypic subpopulations of specialized cells that coexist and have a very efficient mode of communication. These subpopulations sense specific signals that determine a pathway of regulatory events, which in turn triggers specific cell differentiation processes into specific cell fates. Lopez *et al*. [51] described that the triggering of these differentiation processes is mediated by the phosphorylation of three master regulators: DegU, ComK, and Spo0A [52]. Our topological analysis revealed a module devoted to cell differentiation, in which the following well-defined cellular responses and fates were clustered together: *sporulation*, *competence*, *DNA protection*, *matrix and extracellular protein biogenesis*, *cannibalism*, *degradative enzyme synthesis*, *and nutritional limitation response* (Figure 5 and Table 3 supplementary material). This analysis also illustrates the direct and indirect influence of the master transcriptional regulators ArB, Spo0A and ComK [52] on various differentiation processes and stress responses.

On one hand, AbrB (Figure 5) is a TF that is directly connected to different responses such as *competence*, *nutritional limitation response*, *matrix and extracellular proteins biogenesis*, *sporulation*, and *cannibalism.* On the other hand, Spo0A directly regulates the expression of AbrB and other TFs that are involved in *sporulation*, *matrix and extracellular proteins biogenesis*, and *cannibalism*. Other responses, such as *DNA* *protection* and *degradative enzymes*, are connected to the network through ComK, which is directly regulated by AbrB. AbrB also regulates the RNA polymerase σ factor σH, which coordinates the expression of genes related to the *DNA protection* response. The *DNA protection* response is regulated by LexA and Hbs, which are associated with the care, protection, organization, and structuring of DNA. Hbs is required during sporulation, although it is also important during vegetative growth [53]. In contrast, the DNA damage response regulated by LexA can prevent cells from entering sporulation through a mechanism that synchronizes the *B. subtilis* cell cycle with the initiation of sporulation [54]. Cell synchronization guarantees that two complete chromosomes are available before cell division: one chromosome is destined to the pre-spore, and the other is destined for the mother cell. Experiments performed using flow cytometry showed that LexA modulates the expression of *sda,* a checkpoint factor that prevents sporulation in response to DNA damage. Two other TFs participate in this process: DnaA and Spo0A. DNA is a TF with experimentally verified binding sites over the *sda* regulatory region, while Spo0A acts directly as a positive regulator of *sda* transcription [54]. The experiments presented by Errington and colleges [54] showed that sda transcription is promoted by the active form of DnaA and is independently modulated by both Spo0A∼P and LexA. DnaA was classified in our study but only with weak experimental evidence; therefore, it was not included in our *B. subtilis* regulatory network.

Although σH is not considered to be a master regulator, it plays important roles in the modulation of different responses, such as controlling the expression of the *spo0A* in sporulation [37] or interacting with the global regulator AbrB to control different cell fates and cellular responses (see Figure 5).

Here, we present a brief description of the TFs that are involved in each of the main *B. subtilis* responses. Sporulation is the best studied process in this bacterium, and Spo0A is considered to be a master regulator. This TF is phosphorylated when cell density and nutrient starvation are sensed [37]. Subsequently, Spo0A triggers the asymmetric division of the cell and the genetic cascades found in both the mother cell and the forespore [37]. The event in the forespore begins with the expression of *sigF*, which encodes σF; its regulon includes genes that are required for the first events in sporulation, some of which encode regulatory proteins, while others encode proteins that are involved in the morphogenesis or the resistance and germination of the spore [55]. RsfA, another TF included in the cluster, corregulates with σG diverse genes that are involved in the regulatory signal cascade and genes whose products contribute to the proper formation of the spore [55]. Additionally, SpoVT and SplA regulate part of the expression of the σG regulon [45]. In the sporulation group of the *Cell differentiation* module, the transcription regulation depends not only on TFs, but also on the presence of different σ factors that are coordinately and hierarchically expressed (see Figure 5).

In addition to the regulation described above, AbrB mainly regulates genes that have been implicated in the transition from exponential growth to the stationary phase [56] and is fundamental for the development of mutually exclusive cell fates and cellular responses, as shown in Figure 5 [52]. AbrB inhibits sporulation by repressing σH and regulating the expression of proteins that control *extracellular protein biogenesis*, such as the regulators SenS and Hpr, which are included in this module [57, 58]. Hpr is regulated by SalA, which is induced by disulfide stress [59]. Prior to sporulation, *B. subtilis* communities may form biofilms, where some of the cells differentiate into matrix producers. In our analysis, we identified that the genes that are involved in the *matrix and extracellular protein biogenesis response* can also be triggered by Spo0A, although in a lesser-phosphorylated state than is required to activate the *sporulation response*. This phosphorylated state of Spo0A activates the transcription of the *sinIR* operon, which in turn regulates the transition from *independent*, *motile cells* to *matrix-cell producers*. SinR, another TF clustered in this module, represses *matrix formation* (Figure 5). While SinI, the other protein codified in the operon, the other protein codified in the operon, controls SinR activity not at the transcriptional level but by a protein-protein interaction that antagonizes the effect of SinR over the target promoters [60]. *B. subtilis* also differentiates into a cell type known as *cannibals* (Figure 5), in which the cells that have already committed to sporulation delay spore formation by producing toxins that are exported to destroy neighbor cells that have not initiated the sporulation pathway. The production- and exportation-related genes of these toxins are encoded in the *skfABCDGH* and *sdpRI* operons, which are both regulated by Spo0A and AbrB. A third operon that has also been described is *sdpRI*, the expression of which modulates immunity for the bacteriocins [24]. AbrB represses its expression, while Spo0A induces it. This expression pattern means that the cannibalistic cells carry immunity, while the ones that have not expressed Spo0A are susceptible to the toxins [61]. In addition, AbrB represses the expression of FatR, which is involved in the adaptive response to the toxicity of fatty acids [62]. AbrB also inhibits the genes that are involved in another *cell fate* known as *competence* in which corresponding genes in *B. subtilis* are also controlled by two TFs, ComK and Rok. These two TFs are regulated by a complex regulatory network that connects them to other cellular processes or cell fates. For instance, ComK is regulated by four different TFs: Rok regulates *comK* and its own expression; the master regulator DegU controls *degradative enzymes* *synthesis* and the expression of motile cells [63, 64]; Med contributes to the *nutritional limitation* response; and CodY represses the expression of the gene encoding ComK and HutP (Figure 5), a TF that is involved in regulating histidine utilization [65]. Rok regulates genes that are involved in transport systems, antibiotics production, sporulation, and competence [66], and it is both auto-regulated and regulated by SinR, a TF of the *matrix and extracellular protein biogenesis* response.

The *degradative enzymes synthesis* response (Figure 5), is regulated by DegU and the TFs SacT and SacY, which are also involved in sucrose uptake [67]. The activities of these TFs are modulated by reversible phosphorylation, although their regulatory mechanisms are quite different. DegU is the response regulator of a two-component regulatory system and is active when it is phosphorylated by DegS. In contrast, SacY and SacT are RNA-binding paralogous proteins (Figure 3) and members of a family of anti-termination proteins that can be found in two different forms: an inactive monomeric phosphorylated form and an active dimeric nonphosphorylated form. In their active states, these regulators prevent premature transcription termination at the 5’ regions of sacB and sacPA. The phosphorylation state of SacY is regulated by SacX, a sugar phosphotransferase-like protein, in response to sucrose availability in the culture medium [68]. Finally, as shown in Figure 3, σG and σF are paralogous proteins of the cell fate process that are directly devoted to sporulation and participate in pre-spore formation. The above description of this module is a clear example of how our analysis depicts a complex TRN, which allows a dynamic genetic response that depends on intracellular and environmental signals and determines not only the metabolic and structural state of a single cell, but also the different cell fates present in an entire population, ultimately allowing the survival of the community.

**General stress module 9.** In addition to cell differentiation, *B. subtilis* has other methods to face adverse growth conditions. The genes involved in these activities are regulated by different σ factors and TFs that clustered in the *General stress module****.*** T The σB regulon is one of the alternative responses, and it is activated to protect the vegetative cell during starvation or physical stress [69]. This regulon involves the transcription of many genes, including TFs such as CtsR, BmrR, and Spx, which are master regulators of different stress responses (Figure 2). CtsR [70] regulates genes that encode the proteins that are required to repair proteins damaged by heat and oxidative stress, while BmrR regulates antibiotic resistance-related genes [71]. Spx is another important TF clustered in this module that coordinates the adaptation of *B. subtilis* to disulfide stress, resistance to hydrogen peroxide, and the response to sulfur availability [72, 73]. It controls the expression of the σ factor YlaC and the TF YtlI. YtlI is likewise regulated by CymR, a master regulator of sulfur metabolism [74]. Beside σB regulation, Spx is regulated by many other TFs in the module, two of which are directly involved in the resistance to oxidative stress: PerR and YodB [75]. YodB participates in the regulation of the oxidative stress response by modulating the transcription of *yodC* and *spx* [75]. PerR is the master regulator of the peroxide stress response and is a paralogous copy of Fur (Figure 3), an important TF that is involved in iron uptake [76]. Additionally, σM, σW, and σX are three extracytoplasmic σ factors that also regulate the expression of *spx*. The contribution of σM has been described in the response to growth under osmotic stresses, and it is involved in the preservation of membrane and cell wall integrity in response to stresses such as ethanol, heat-shock, acid, and superoxide stress (Figure 2) [77]. In another context, σW regulates the expression of proteins that defend the cell against antimicrobial compounds [78]. Lastly, σX regulates LytR, a TF clustered in this module that regulates the transcription of genes that are involved in the secretion system of autolysins [49]. In turn, σX is regulated by YvrH, a TF involved in the homeostasis of *B. subtilis* at the cell surface level.

**Table S2. Functional descriptions of the transcription and σ factors that were included in diverse modules.** The table provides a brief functional description of the regulatory proteins and σ factors that were organized in each module.

| **Module Name** | **Regulatory gene** | **Regulatory function** | **Reference** |
| --- | --- | --- | --- |
| Nucleotide synthesis | Purr | Metabolism, synthesis and transport of purines | [5] |
| Nucleotide synthesis | pyrR | Regulation of the pyrimidine biosynthetic operon (*pyr*) | [4, 79] |
| Degradative enzymes | tenA | May indirectly enhance the production of extracellular enzymes | [8] |
| Degradative enzymes | tenI | May directly bind to the promoter of *aprE* or may modify *tenA* through chemical modifications or the complex formation | [8] |
| Secretion | htrA | Responds to secretion stress and heat shock in a manner dependent on the CssRS two-component system | [80, 81] |
| Secretion | cssR | Responds to stress caused by the secretion and accumulation of misfolded proteins located between the membrane and cell wall | [7, 81] |
| Nitrogen metabolism | tnrA | Regulates the expression of gene in nitrogen-limited growth | [2, 82] |
| Nitrogen metabolism | glnR | Excess nitrogen regulates the synthesis of glutamine, glutamate, urease, and the *tnrA* gene | [2] |
| Nitrogen metabolism | kipR | Represses the expression of the histidine kinases KipA and KipI | [2, 3] |
| General stress | yvrH | Regulates homeostasis at the cell surface level | [49] |
| General stress | lytR | Modulates the expression of the genes *lytABC* encoding N-acetylmuramoyl-L-alanine amidase, related to the secretion system of autolysins | [83] |
| General stress | ytlI | Regulates the expression of genes involved in sulfur metabolism | [84] |
| General stress | ylaC | An extracytoplasmatic σ factor; contributes to hydrogen peroxide resistance | [73] |
| General stress | sigW | Associated with detoxification function and protects against antimicrobials | [85] |
| General stress | sigX | Regulates genes related to metabolism and cell wall composition | [86] |
| General stress | ctsR | Regulates the expression of genes involved in protein renaturation, protein repair, and ATP-dependent proteolysis | [70, 87] |
| General stress | spx | Also known as YjbD; responds to disulfide stress | [88] |
| General stress | mta | Responds to stress caused by the secretion and accumulation of misfolded proteins located between the membrane and cell wall | [89] |
| General stress | yrzC | Also known as CymR; is the master regulator of sulfur metabolism | [74] |
| General stress | fur | Regulates genes implicated in siderophore synthesis; also associated with iron transport | [76] |
| General stress | perR | Transcription induced by hydrogen peroxide, general stress, or entry into the stationary phase in response to iron and manganese limitation | [90, 76] |
| General stress | bmrR | Multidrug-responding transcriptional regulator; induced by rhodamine and tetraphenylphosphonium | [71, 91] |
| General stress | sigB | Involved in multiple stress-responsive, non-specific and preventive | [92, 93] |
| Respiration | Fnr | Regulatory protein; part of the regulatory cascade that controls the adaptation to anaerobic growth caused by low oxygen tension | [94, 95] |
| Respiration | resD | Plays a global role in both aerobic and anaerobic respiration | [96] |
| Respiration | phoP | Responds to low concentrations of inorganic phosphate | [97] |
| Respiration | hrcA | Induced by heat-shock stress | [98] |
| Respiration | arfM | Regulates anaerobic and fermentative growth | [99] |
| Respiration | nsrR | Regulates genes involved in anaerobiosis when nitric oxide is present | [10] |
| Cascade of the mother cell sporulating module | yfhP | May act as a negative regulator for the transcription of yfhQ, yfhR, *sspE*, and *yfhP*; regulated by *sigK*, a protein that is regulated by the expression of genes used in the mother cell compartment | [48, 50] |
| Cascade of the mother cell sporulating module | gerR | Directly or indirectly regulates the expression of several late sporulation genes | [100] |
| Cascade of the mother cell sporulating module | sigK | Regulates gene expression in late mother cell; directs the transcription of genes encoding the most abundant proteins of the spore coat | [101] |
| Cascade of the mother cell sporulating module | sigE | Regulates gene expressing in early mother cell | [101] |
| Cascade of the mother cell sporulating module | gerE | Regulates the transcription of many genes in the mother cell during the late stages of sporulation | [47] |
| Cascade of the mother cell sporulating module | spoIIID | A bi-functional transcription factor; regulates temporal expression of many genes in the mother cell | [35] |
| Carbon metabolism | glvR | Controls the expression of genes that catabolizes maltose | [22] |
| Carbon metabolism | acoR | Positively regulates the acetoin dehydrogenase operon *acoABCL* | [19] |
| Carbon metabolism | gntR | Negatively regulates gluconate-inducible expression of the *gntRKPZ* operon | [16, 102] |
| Carbon metabolism | exuR | Controls the expression of the  *uxaC-yjmBCD-uxuA-yjmF-exuTR-uxaBA* operon, which is induced by galacturonate | [18] |
| Carbon metabolism | fadR | Regulates the operon involved in fatty acids degradation | [23] |
| Carbon metabolism | treR | Regulates the *trePAR* operon that is involved in trehalose catabolism | [21] |
| Carbon metabolism | ccpB | Mediates catabolic repression by glucose, mannitol, and sucrose of the gluconate (gnt) and xylose (xyl) operons, acts in parallel with CcpA | [103] |
| Carbon metabolism | sigL | Regulates genes that participate in the metabolism of fructose, levanase, arginine, and acetoin | [104] |
| Carbon metabolism | ccpC | Exerts catabolic repression of genes encoding enzymes involved in the Krebs cycle | [30] |
| Carbon metabolism | ccpA | Master regulator of carbon catabolism | [105] |
| Cell differentiation | hbs or hbsU | Histone-like proteins; may wrap the DNA and condense the chromosome into highly folded nucleoid structures | [106] |
| Cell differentiation | lexA | Negatively regulates DNA damage-inducible genes; involve in the SOS response; analog of *E. coli* LexA | [107] |
| Cell differentiation | sigG | Directs transcription of genes that are expressed only in the forespore | [108] |
| Cell differentiation | spoVT | Controls σG-dependent gene expression | [109] |
| Cell differentiation | sacT | Regulates sucrose utilization and the production of levansucrase | [110] |
| Cell differentiation | sigH | Directs transcription of genes whose products redirect septum formation from the mid-cell to a polar position and partition one copy of the chromosome to the larger mother cell and the other copy to the smaller forespore. | [111] |
| Cell differentiation | sigF | RNA polymerase sporulation-specific σ factor | [112] |
| Cell differentiation | splA | Activates the expression of *slpAB* operon during germination | [113] |
| Cell differentiation | hpr | Used during the transition state between vegetative growth and the onset of sporulation | [114] |
| Cell differentiation | sdpR | Modulates immunity for the bacteriocins | [24] |
| Cell differentiation | hutP | Involved in histidine utilization | [115] |
| Cell differentiation | sinR | Essential for the late-growth processes of competence and motility and also represses others processes (e.g., sporulation and subtilisin synthesis) | [116] |
| Cell differentiation | salA | Induced by disulfide stress | [59] |
| Cell differentiation | fatR | Involved in the adaptive response to the toxicity of fatty acids | [62] |
| Cell differentiation | rsfA | Represses the expression spoIIR*, a signaling gene that is involved in triggering the appearance of σ^E^* in the mother cell. | [117, 55] |
| Cell differentiation | sacY | Controls the expression of genes and operons required for the utilization of sucrose and levansucrase | [110] |
| Cell differentiation | comK | Induces competence | [118] |
| Cell differentiation | med | Disruption leads to significant decrease of *comK*, expression | [119] |
| Cell differentiation | degU | Is the master controller of extracellular enzyme production and expression in motile cells | [110] |
| Cell differentiation | codY | Regulates genes that are involved in transport systems, antibiotic production, sporulation, and competence | [65] |
| Cell differentiation | spo0A | Is the master regulator of sporulation | [120] |
| Cell differentiation | abrB | Bi-functional; expressed during the transition state between vegetative growth and the onset of the stationary phase and sporulation; acts in the Spo0A-AbrB circuit; also involved in catabolic repression | [58] |

**Reference List**

**1. Martinez-Nunez MA, Perez-Rueda E, Gutierrez-Rios RM, Merino E: New insights into the regulatory networks of paralogous genes in bacteria. *Microbiology* 2010, 156:14-22.**

**2. Doroshchuk NA, Gel'fand MS, Rodionov DA: [Regulation of nitrogen metabolism in gram-positive bacteria]. *Mol Biol (Mosk)* 2006, 40:919-926.**

**3. Wang L, Grau R, Perego M, Hoch JA: A novel histidine kinase inhibitor regulating development in Bacillus subtilis. *Genes Dev* 1997, 11:2569-2579.**

**4. Turner RJ, Lu Y, Switzer RL: Regulation of the Bacillus subtilis pyrimidine biosynthetic (pyr) gene cluster by an autogenous transcriptional attenuation mechanism. *J Bacteriol* 1994, 176:3708-3722.**

**5. Bera AK, Zhu J, Zalkin H, Smith JL: Functional dissection of the Bacillus subtilis pur operator site. *J Bacteriol* 2003, 185:4099-4109.**

**6. Smith E, Morowitz HJ: Universality in intermediary metabolism. *Proc Natl Acad Sci U S A* 2004, 101:13168-13173.**

**7. Hyyrylainen HL, Bolhuis A, Darmon E, Muukkonen L, Koski P, Vitikainen M, Sarvas M, Pragai Z, Bron S, van Dijl JM, Kontinen VP: A novel two-component regulatory system in Bacillus subtilis for the survival of severe secretion stress. *Mol Microbiol* 2001, 41:1159-1172.**

**8. Pang AS, Nathoo S, Wong SL: Cloning and characterization of a pair of novel genes that regulate production of extracellular enzymes in Bacillus subtilis. *J Bacteriol* 1991, 173:46-54.**

**9. Nakano MM, Zuber P: Anaerobic growth of a "strict aerobe" (Bacillus subtilis). *Annu Rev Microbiol* 1998, 52:165-190.**

**10. Nakano MM, Geng H, Nakano S, Kobayashi K: The nitric oxide-responsive regulator NsrR controls ResDE-dependent gene expression. *J Bacteriol* 2006, 188:5878-5887.**

**11. Sun G, Birkey SM, Hulett FM: Three two-component signal-transduction systems interact for Pho regulation in Bacillus subtilis. *Mol Microbiol* 1996, 19:941-948.**

**12. Birkey SM, Liu W, Zhang X, Duggan MF, Hulett FM: Pho signal transduction network reveals direct transcriptional regulation of one two-component system by another two-component regulator: Bacillus subtilis PhoP directly regulates production of ResD. *Mol Microbiol* 1998, 30:943-953.**

**13. Gorke B, Stulke J: Carbon catabolite repression in bacteria: many ways to make the most out of nutrients. *Nat Rev Microbiol* 2008, 6:613-624.**

**14. Stulke J, Hillen W: Regulation of carbon catabolism in Bacillus species. *Annu Rev Microbiol* 2000, 54:849-880.**

**15. Miwa Y, Nakata A, Ogiwara A, Yamamoto M, Fujita Y: Evaluation and characterization of catabolite-responsive elements (cre) of Bacillus subtilis. *Nucleic Acids Res* 2000, 28:1206-1210.**

**16. Fujita Y, Fujita T, Miwa Y, Nihashi J, Aratani Y: Organization and transcription of the gluconate operon, gnt, of Bacillus subtilis. *J Biol Chem* 1986, 261:13744-13753.**

**17. Fujita Y, Miwa Y: Catabolite repression of the Bacillus subtilis gnt operon mediated by the CcpA protein. *J Bacteriol* 1994, 176:511-513.**

**18. Mekjian KR, Bryan EM, Beall BW, Moran CP, Jr.: Regulation of hexuronate utilization in Bacillus subtilis. *J Bacteriol* 1999, 181:426-433.**

**19. Ali NO, Bignon J, Rapoport G, Debarbouille M: Regulation of the acetoin catabolic pathway is controlled by sigma L in Bacillus subtilis. *J Bacteriol* 2001, 183:2497-2504.**

**20. Huang M, Oppermann-Sanio FB, Steinbuchel A: Biochemical and molecular characterization of the Bacillus subtilis acetoin catabolic pathway. *J Bacteriol* 1999, 181:3837-3841.**

**21. Schock F, Dahl MK: Expression of the tre operon of Bacillus subtilis 168 is regulated by the repressor TreR. *J Bacteriol* 1996, 178:4576-4581.**

**22. Yamamoto H, Serizawa M, Thompson J, Sekiguchi J: Regulation of the glv operon in Bacillus subtilis: YfiA (GlvR) is a positive regulator of the operon that is repressed through CcpA and cre. *J Bacteriol* 2001, 183:5110-5121.**

**23. Tojo S, Satomura T, Matsuoka H, Hirooka K, Fujita Y: Catabolite repression of the Bacillus subtilis FadR regulon, which is involved in fatty acid catabolism. *J Bacteriol* 2011, 193:2388-2395.**

**24. Gonzalez-Pastor JE, Hobbs EC, Losick R: Cannibalism by sporulating bacteria. *Science* 2003, 301:510-513.**

**25. Hueck CJ, Hillen W: Catabolite repression in Bacillus subtilis: a global regulatory mechanism for the gram-positive bacteria? *Mol Microbiol* 1995, 15:395-401.**

**26. Debarbouille M, Martin-Verstraete I, Kunst F, Rapoport G: The Bacillus subtilis sigL gene encodes an equivalent of sigma 54 from gram-negative bacteria. *Proc Natl Acad Sci U S A* 1991, 88:9092-9096.**

**27. Gardan R, Rapoport G, Debarbouille M: Role of the transcriptional activator RocR in the arginine-degradation pathway of Bacillus subtilis. *Mol Microbiol* 1997, 24:825-837.**

**28. Debarbouille M, Gardan R, Arnaud M, Rapoport G: Role of bkdR, a transcriptional activator of the sigL-dependent isoleucine and valine degradation pathway in Bacillus subtilis. *J Bacteriol* 1999, 181:2059-2066.**

**29. Wiegeshoff F, Beckering CL, Debarbouille M, Marahiel MA: Sigma L is important for cold shock adaptation of Bacillus subtilis. *J Bacteriol* 2006, 188:3130-3133.**

**30. Kim SI, Jourlin-Castelli C, Wellington SR, Sonenshein AL: Mechanism of repression by Bacillus subtilis CcpC, a LysR family regulator. *J Mol Biol* 2003, 334:609-624.**

**31. Kallio PT, Fagelson JE, Hoch JA, Strauch MA: The transition state regulator Hpr of Bacillus subtilis is a DNA-binding protein. *J Biol Chem* 1991, 266:13411-13417.**

**32. Kim HJ, Jourlin-Castelli C, Kim SI, Sonenshein AL: Regulation of the bacillus subtilis ccpC gene by ccpA and ccpC. *Mol Microbiol* 2002, 43:399-410.**

**33. Blencke HM, Reif I, Commichau FM, Detsch C, Wacker I, Ludwig H, Stulke J: Regulation of citB expression in Bacillus subtilis: integration of multiple metabolic signals in the citrate pool and by the general nitrogen regulatory system. *Arch Microbiol* 2006, 185:136-146.**

**34. Wall ME, Dunlop MJ, Hlavacek WS: Multiple functions of a feed-forward-loop gene circuit. *J Mol Biol* 2005, 349:501-514.**

**35. Eichenberger P, Fujita M, Jensen ST, Conlon EM, Rudner DZ, Wang ST, Ferguson C, Haga K, Sato T, Liu JS, Losick R: The program of gene transcription for a single differentiating cell type during sporulation in Bacillus subtilis. *PLoS Biol* 2004, 2:e328.**

**36. Kroos L, Zhang B, Ichikawa H, Yu YT: Control of sigma factor activity during Bacillus subtilis sporulation. *Mol Microbiol* 1999, 31:1285-1294.**

**37. Fujita M, Gonzalez-Pastor JE, Losick R: High- and low-threshold genes in the Spo0A regulon of Bacillus subtilis. *J Bacteriol* 2005, 187:1357-1368.**

**38. Eichenberger P, Jensen ST, Conlon EM, van OC, Silvaggi J, Gonzalez-Pastor JE, Fujita M, Ben-Yehuda S, Stragier P, Liu JS, Losick R: The sigmaE regulon and the identification of additional sporulation genes in Bacillus subtilis. *J Mol Biol* 2003, 327:945-972.**

**39. Henriques AO, Moran CP, Jr.: Structure, assembly, and function of the spore surface layers. *Annu Rev Microbiol* 2007, 61:555-588.**

**40. Homuth G, Heinemann M, Zuber U, Schumann W: The genes of lepA and hemN form a bicistronic operon in Bacillus subtilis. *Microbiology* 1996, 142 ( Pt 7):1641-1649.**

**41. Kunkel B, Kroos L, Poth H, Youngman P, Losick R: Temporal and spatial control of the mother-cell regulatory gene spoIIID of Bacillus subtilis. *Genes Dev* 1989, 3:1735-1744.**

**42. Feucht A, Evans L, Errington J: Identification of sporulation genes by genome-wide analysis of the sigmaE regulon of Bacillus subtilis. *Microbiology* 2003, 149:3023-3034.**

**43. Guillot C, Moran CP, Jr.: Essential internal promoter in the spoIIIA locus of Bacillus subtilis. *J Bacteriol* 2007, 189:7181-7189.**

**44. Chastanet A, Losick R: Engulfment during sporulation in Bacillus subtilis is governed by a multi-protein complex containing tandemly acting autolysins. *Mol Microbiol* 2007, 64:139-152.**

**45. Hilbert DW, Piggot PJ: Compartmentalization of gene expression during Bacillus subtilis spore formation. *Microbiol Mol Biol Rev* 2004, 68:234-262.**

**46. Cutting S, Panzer S, Losick R: Regulatory studies on the promoter for a gene governing synthesis and assembly of the spore coat in Bacillus subtilis. *J Mol Biol* 1989, 207:393-404.**

**47. Zheng L, Halberg R, Roels S, Ichikawa H, Kroos L, Losick R: Sporulation regulatory protein GerE from Bacillus subtilis binds to and can activate or repress transcription from promoters for mother-cell-specific genes. *J Mol Biol* 1992, 226:1037-1050.**

**48. Fujita M: Identification of new sigma K-dependent promoters using an in vitro transcription system derived from Bacillus subtilis. *Gene* 1999, 237:45-52.**

**49. Serizawa M, Kodama K, Yamamoto H, Kobayashi K, Ogasawara N, Sekiguchi J: Functional analysis of the YvrGHb two-component system of Bacillus subtilis: identification of the regulated genes by DNA microarray and northern blot analyses. *Biosci Biotechnol Biochem* 2005, 69:2155-2169.**

**50. Yamamoto H, Mori M, Sekiguchi J: Transcription of genes near the sspE locus of the Bacillus subtilis genome. *Microbiology* 1999, 145 ( Pt 8):2171-2180.**

**51. Lopez D, Kolter R: Extracellular signals that define distinct and coexisting cell fates in Bacillus subtilis. *FEMS Microbiol Rev* 2010, 34:134-149.**

**52. Lopez D, Vlamakis H, Kolter R: Generation of multiple cell types in Bacillus subtilis. *FEMS Microbiol Rev* 2009, 33:152-163.**

**53. Micka B, Marahiel MA: The DNA-binding protein HBsu is essential for normal growth and development in Bacillus subtilis. *Biochimie* 1992, 74:641-650.**

**54. Veening JW, Murray H, Errington J: A mechanism for cell cycle regulation of sporulation initiation in Bacillus subtilis. *Genes Dev* 2009, 23:1959-1970.**

**55. Wang ST, Setlow B, Conlon EM, Lyon JL, Imamura D, Sato T, Setlow P, Losick R, Eichenberger P: The forespore line of gene expression in Bacillus subtilis. *J Mol Biol* 2006, 358:16-37.**

**56. Moreno-Campuzano S, Janga SC, Perez-Rueda E: Identification and analysis of DNA-binding transcription factors in Bacillus subtilis and other Firmicutes--a genomic approach. *BMC Genomics* 2006, 7:147.**

**57. McCready P, Takagi M, Doi RH: Regulation of Bacillus subtilis senS by homologous regulatory regions of senS and the inducible cat gene. *Biochem Biophys Res Commun* 1993, 193:1110-1115.**

**58. Strauch MA, Spiegelman GB, Perego M, Johnson WC, Burbulys D, Hoch JA: The transition state transcription regulator abrB of Bacillus subtilis is a DNA binding protein. *EMBO J* 1989, 8:1615-1621.**

**59. Leichert LI, Scharf C, Hecker M: Global characterization of disulfide stress in Bacillus subtilis. *J Bacteriol* 2003, 185:1967-1975.**

**60. Bai U, Mandic-Mulec I, Smith I: SinI modulates the activity of SinR, a developmental switch protein of Bacillus subtilis, by protein-protein interaction. *Genes Dev* 1993, 7:139-148.**

**61. Ellermeier CD, Hobbs EC, Gonzalez-Pastor JE, Losick R: A three-protein signaling pathway governing immunity to a bacterial cannibalism toxin. *Cell* 2006, 124:549-559.**

**62. Gustafsson MC, von WC: A novel diffusible substance can overcome the apparent AbrB repression of the Bacillus subtilis fatR promoter. *FEMS Microbiol Lett* 2001, 199:197-202.**

**63. Kunst F, Msadek T, Bignon J, Rapoport G: The DegS/DegU and ComP/ComA two-component systems are part of a network controlling degradative enzyme synthesis and competence in Bacillus subtilis. *Res Microbiol* 1994, 145:393-402.**

**64. Tsukahara K, Ogura M: Promoter selectivity of the Bacillus subtilis response regulator DegU, a positive regulator of the fla/che operon and sacB. *BMC Microbiol* 2008, 8:8.**

**65. Fisher SH, Rohrer K, Ferson AE: Role of CodY in regulation of the Bacillus subtilis hut operon. *J Bacteriol* 1996, 178:3779-3784.**

**66. Albano M, Smits WK, Ho LT, Kraigher B, Mandic-Mulec I, Kuipers OP, Dubnau D: The Rok protein of Bacillus subtilis represses genes for cell surface and extracellular functions. *J Bacteriol* 2005, 187:2010-2019.**

**67. Tortosa P, Le CD: A ribonucleic antiterminator sequence (RAT) and a distant palindrome are both involved in sucrose induction of the Bacillus subtilis sacXY regulatory operon. *Microbiology* 1995, 141 ( Pt 11):2921-2927.**

**68. Crutz AM, Steinmetz M, Aymerich S, Richter R, Le CD: Induction of levansucrase in Bacillus subtilis: an antitermination mechanism negatively controlled by the phosphotransferase system. *J Bacteriol* 1990, 172:1043-1050.**

**69. Petersohn A, Brigulla M, Haas S, Hoheisel JD, Volker U, Hecker M: Global analysis of the general stress response of Bacillus subtilis. *J Bacteriol* 2001, 183:5617-5631.**

**70. Derre I, Rapoport G, Msadek T: CtsR, a novel regulator of stress and heat shock response, controls clp and molecular chaperone gene expression in gram-positive bacteria. *Mol Microbiol* 1999, 31:117-131.**

**71. Ahmed M, Borsch CM, Taylor SS, Vazquez-Laslop N, Neyfakh AA: A protein that activates expression of a multidrug efflux transporter upon binding the transporter substrates. *J Biol Chem* 1994, 269:28506-28513.**

**72. Coppee JY, Auger S, Turlin E, Sekowska A, Le Caer JP, Labas V, Vagner V, Danchin A, Martin-Verstraete I: Sulfur-limitation-regulated proteins in Bacillus subtilis: a two-dimensional gel electrophoresis study. *Microbiology* 2001, 147:1631-1640.**

**73. Ryu HB, Shin I, Yim HS, Kang SO: YlaC is an extracytoplasmic function (ECF) sigma factor contributing to hydrogen peroxide resistance in Bacillus subtilis. *J Microbiol* 2006, 44:206-216.**

**74. Even S, Burguiere P, Auger S, Soutourina O, Danchin A, Martin-Verstraete I: Global control of cysteine metabolism by CymR in Bacillus subtilis. *J Bacteriol* 2006, 188:2184-2197.**

**75. Leelakriangsak M, Kobayashi K, Zuber P: Dual negative control of spx transcription initiation from the P3 promoter by repressors PerR and YodB in Bacillus subtilis. *J Bacteriol* 2007, 189:1736-1744.**

**76. Fuangthong M, Herbig AF, Bsat N, Helmann JD: Regulation of the Bacillus subtilis fur and perR genes by PerR: not all members of the PerR regulon are peroxide inducible. *J Bacteriol* 2002, 184:3276-3286.**

**77. Thackray PD, Moir A: SigM, an extracytoplasmic function sigma factor of Bacillus subtilis, is activated in response to cell wall antibiotics, ethanol, heat, acid, and superoxide stress. *J Bacteriol* 2003, 185:3491-3498.**

**78. Gruber TM, Gross CA: Multiple sigma subunits and the partitioning of bacterial transcription space. *Annu Rev Microbiol* 2003, 57:441-466.**

**79. Zhang H, Switzer RL: Transcriptional pausing in the Bacillus subtilis pyr operon in vitro: a role in transcriptional attenuation? *J Bacteriol* 2003, 185:4764-4771.**

**80. Darmon E, Noone D, Masson A, Bron S, Kuipers OP, Devine KM, van Dijl JM: A novel class of heat and secretion stress-responsive genes is controlled by the autoregulated CssRS two-component system of Bacillus subtilis. *J Bacteriol* 2002, 184:5661-5671.**

**81. Noone D, Howell A, Devine KM: Expression of ykdA, encoding a Bacillus subtilis homologue of HtrA, is heat shock inducible and negatively autoregulated. *J Bacteriol* 2000, 182:1592-1599.**

**82. Kayumov A, Heinrich A, Sharipova M, Iljinskaya O, Forchhammer K: Inactivation of the general transcription factor TnrA in Bacillus subtilis by proteolysis. *Microbiology* 2008, 154:2348-2355.**

**83. Lazarevic V, Margot P, Soldo B, Karamata D: Sequencing and analysis of the Bacillus subtilis lytRABC divergon: a regulatory unit encompassing the structural genes of the N-acetylmuramoyl-L-alanine amidase and its modifier. *J Gen Microbiol* 1992, 138:1949-1961.**

**84. Burguiere P, Fert J, Guillouard I, Auger S, Danchin A, Martin-Verstraete I: Regulation of the Bacillus subtilis ytmI operon, involved in sulfur metabolism. *J Bacteriol* 2005, 187:6019-6030.**

**85. Huang X, Gaballa A, Cao M, Helmann JD: Identification of target promoters for the Bacillus subtilis extracytoplasmic function sigma factor, sigma W. *Mol Microbiol* 1999, 31:361-371.**

**86. Huang X, Fredrick KL, Helmann JD: Promoter recognition by Bacillus subtilis sigmaW: autoregulation and partial overlap with the sigmaX regulon. *J Bacteriol* 1998, 180:3765-3770.**

**87. Kruger E, Hecker M: The first gene of the Bacillus subtilis clpC operon, ctsR, encodes a negative regulator of its own operon and other class III heat shock genes. *J Bacteriol* 1998, 180:6681-6688.**

**88. Erwin KN, Nakano S, Zuber P: Sulfate-dependent repression of genes that function in organosulfur metabolism in Bacillus subtilis requires Spx. *J Bacteriol* 2005, 187:4042-4049.**

**89. Baranova NN, Danchin A, Neyfakh AA: Mta, a global MerR-type regulator of the Bacillus subtilis multidrug-efflux transporters. *Mol Microbiol* 1999, 31:1549-1559.**

**90. Faulkner MJ, Ma Z, Fuangthong M, Helmann JD: Derepression of the Bacillus subtilis PerR peroxide stress response leads to iron deficiency. *J Bacteriol* 2012, 194:1226-1235.**

**91. Markham PN, Ahmed M, Neyfakh AA: The drug-binding activity of the multidrug-responding transcriptional regulator BmrR resides in its C-terminal domain. *J Bacteriol* 1996, 178:1473-1475.**

**92. Hecker M, Pane-Farre J, Volker U: SigB-dependent general stress response in Bacillus subtilis and related gram-positive bacteria. *Annu Rev Microbiol* 2007, 61:215-236.**

**93. Nannapaneni P, Hertwig F, Depke M, Hecker M, Mader U, Volker U, Steil L, van Hijum SA: Defining the structure of the general stress regulon of Bacillus subtilis using targeted microarray analysis and random forest classification. *Microbiology* 2012, 158:696-707.**

**94. Reents H, Gruner I, Harmening U, Bottger LH, Layer G, Heathcote P, Trautwein AX, Jahn D, Hartig E: Bacillus subtilis Fnr senses oxygen via a [4Fe-4S] cluster coordinated by three cysteine residues without change in the oligomeric state. *Mol Microbiol* 2006, 60:1432-1445.**

**95. Reents H, Munch R, Dammeyer T, Jahn D, Hartig E: The Fnr regulon of Bacillus subtilis. *J Bacteriol* 2006, 188:1103-1112.**

**96. Geng H, Zhu Y, Mullen K, Zuber CS, Nakano MM: Characterization of ResDE-dependent fnr transcription in Bacillus subtilis. *J Bacteriol* 2007, 189:1745-1755.**

**97. Allenby NE, O'Connor N, Pragai Z, Ward AC, Wipat A, Harwood CR: Genome-wide transcriptional analysis of the phosphate starvation stimulon of Bacillus subtilis. *J Bacteriol* 2005, 187:8063-8080.**

**98. Schulz A, Schumann W: hrcA, the first gene of the Bacillus subtilis dnaK operon encodes a negative regulator of class I heat shock genes. *J Bacteriol* 1996, 178:1088-1093.**

**99. Marino M, Ramos HC, Hoffmann T, Glaser P, Jahn D: Modulation of anaerobic energy metabolism of Bacillus subtilis by arfM (ywiD). *J Bacteriol* 2001, 183:6815-6821.**

**100. Cangiano G, Mazzone A, Baccigalupi L, Isticato R, Eichenberger P, De FM, Ricca E: Direct and indirect control of late sporulation genes by GerR of Bacillus subtilis. *J Bacteriol* 2010, 192:3406-3413.**

**101. Losick R, Stragier P: Crisscross regulation of cell-type-specific gene expression during development in B. subtilis. *Nature* 1992, 355:601-604.**

**102. Miwa Y, Fujita Y: Purification and characterization of a repressor for the Bacillus subtilis gnt operon. *J Biol Chem* 1988, 263:13252-13257.**

**103. Chauvaux S, Paulsen IT, Saier MH, Jr.: CcpB, a novel transcription factor implicated in catabolite repression in Bacillus subtilis. *J Bacteriol* 1998, 180:491-497.**

**104. Choi SK, Saier MH, Jr.: Regulation of sigL expression by the catabolite control protein CcpA involves a roadblock mechanism in Bacillus subtilis: potential connection between carbon and nitrogen metabolism. *J Bacteriol* 2005, 187:6856-6861.**

**105. Marciniak BC, Pabijaniak M, de JA, Duhring R, Seidel G, Hillen W, Kuipers OP: High- and low-affinity cre boxes for CcpA binding in Bacillus subtilis revealed by genome-wide analysis. *BMC Genomics* 2012, 13:401.**

**106. Micka B, Groch N, Heinemann U, Marahiel MA: Molecular cloning, nucleotide sequence, and characterization of the Bacillus subtilis gene encoding the DNA-binding protein HBsu. *J Bacteriol* 1991, 173:3191-3198.**

**107. Au N, Kuester-Schoeck E, Mandava V, Bothwell LE, Canny SP, Chachu K, Colavito SA, Fuller SN, Groban ES, Hensley LA, O'Brien TC, Shah A, Tierney JT, Tomm LL, O'Gara TM, Goranov AI, Grossman AD, Lovett CM: Genetic composition of the Bacillus subtilis SOS system. *J Bacteriol* 2005, 187:7655-7666.**

**108. Nicholson WL, Sun DX, Setlow B, Setlow P: Promoter specificity of sigma G-containing RNA polymerase from sporulating cells of Bacillus subtilis: identification of a group of forespore-specific promoters. *J Bacteriol* 1989, 171:2708-2718.**

**109. Bagyan I, Hobot J, Cutting S: A compartmentalized regulator of developmental gene expression in Bacillus subtilis. *J Bacteriol* 1996, 178:4500-4507.**

**110. Crutz AM, Steinmetz M: Transcription of the Bacillus subtilis sacX and sacY genes, encoding regulators of sucrose metabolism, is both inducible by sucrose and controlled by the DegS-DegU signalling system. *J Bacteriol* 1992, 174:6087-6095.**

**111. Saujet L, Monot M, Dupuy B, Soutourina O, Martin-Verstraete I: The key sigma factor of transition phase, SigH, controls sporulation, metabolism, and virulence factor expression in Clostridium difficile. *J Bacteriol* 2011, 193:3186-3196.**

**112. Decatur A, Losick R: Identification of additional genes under the control of the transcription factor sigma F of Bacillus subtilis. *J Bacteriol* 1996, 178:5039-5041.**

**113. Fajardo-Cavazos P, Nicholson WL: The TRAP-like SplA protein is a trans-acting negative regulator of spore photoproduct lyase synthesis during Bacillus subtilis sporulation. *J Bacteriol* 2000, 182:555-560.**

**114. Strauch MA, Hoch JA: Transition-state regulators: sentinels of Bacillus subtilis post-exponential gene expression. *Mol Microbiol* 1993, 7:337-342.**

**115. Kumarevel T: Structural insights of HutP-mediated regulation of transcription of the hut operon in Bacillus subtilis. *Biophys Chem* 2007, 128:1-12.**

**116. Chu F, Kearns DB, Branda SS, Kolter R, Losick R: Targets of the master regulator of biofilm formation in Bacillus subtilis. *Mol Microbiol* 2006, 59:1216-1228.**

**117. Juan WL, Errington J: Identification and characterization of a new prespore-specific regulatory gene, rsfA, of Bacillus subtilis. *J Bacteriol* 2000, 182:418-424.**

**118. van SD, Venema G: comK acts as an autoregulatory control switch in the signal transduction route to competence in Bacillus subtilis. *J Bacteriol* 1994, 176:5762-5770.**

**119. Ogura M, Ohshiro Y, Hirao S, Tanaka T: A new Bacillus subtilis gene, med, encodes a positive regulator of comK. *J Bacteriol* 1997, 179:6244-6253.**

**120. Molle V, Fujita M, Jensen ST, Eichenberger P, Gonzalez-Pastor JE, Liu JS, Losick R: The Spo0A regulon of Bacillus subtilis. *Mol Microbiol* 2003, 50:1683-1701.**
